# Death‐Associated Protein 3 Triggers Intrinsic Apoptosis via Miro1 Upon Inducing Intracellular Calcium Changes

**DOI:** 10.1002/mco2.70214

**Published:** 2025-05-10

**Authors:** Dongxue Hu, Qiaoyun Yang, Hongxu Xian, Minghao Wang, Hong Zheng, Karthik Babu Mallilankaraman, Victor C. Yu, Yih‐Cherng Liou

**Affiliations:** ^1^ Department of Biological Sciences Faculty of Science National University of Singapore Singapore Singapore; ^2^ Department of Pharmacology School of Medicine University of California San Diego La Jolla California USA; ^3^ Department of Breast and Thyroid Surgery Southwest Hospital Army Medical University Chongqing China; ^4^ Department of Thoracic Surgery Xinqiao Hospital Army Medical University Chongqing China; ^5^ Department of Physiology Perelman School of Medicine University of Pennsylvania Pennsylvania USA; ^6^ The Fifth Affiliated Hospital of Zhengzhou University Zhengzhou China; ^7^ Tianjian Laboratory of Advanced Biomedical Sciences Zhengzhou China; ^8^ School of Life Sciences Zhengzhou University Zhengzhou China; ^9^ Integrative Sciences and Engineering Programme NUS Graduate School, National University of Singapore Singapore Singapore

**Keywords:** calcium, cell death, death‐associated protein 3, mitochondrial dynamics, reactive oxygen species

## Abstract

Mitochondrial homeostasis is essential for cell survival and function, necessitating quality control mechanisms to ensure a healthy mitochondrial network. Death‐associated protein 3 (DAP3) serves as a subunit of the mitochondrial ribosome, playing a pivotal role in the translation of mitochondrial‐encoded proteins. Apart from its involvement in protein synthesis, DAP3 has been implicated in the process of cell death and mitochondrial dynamics. In this study, we demonstrate that DAP3 mediates cell death via intrinsic apoptosis by triggering excessive mitochondrial fragmentation, loss of mitochondrial membrane potential (Δ*Ψ*m), ATP decline, and oxidative stress. Notably, DAP3 induces mitochondrial fragmentation through the Mitochondrial Rho GTPase 1 (Miro1), independently of the canonical fusion/fission machinery. Mechanistically, DAP3 promotes mitochondrial calcium accumulation through the MCU complex, leading to decreased cytosolic Ca^2+^ levels. This reduction in cytosolic Ca^2+^ is sensed by Miro1, which subsequently drives mitochondrial fragmentation. Depletion of Miro1 or MCU alleviates mitochondrial fragmentation, oxidative stress, and cell death. Collectively, our findings reveal a novel function of the mitoribosomal protein DAP3 in regulating calcium signalling and maintaining mitochondrial homeostasis.

## Graphical abstract

DAP3 regulates intracellular Ca^2+^ via the MCU complex. Upregulated level of DAP3 promotes mitochondrial calcium uptake, leading to decreased cytosolic Ca^2+^ levels ([Ca^2+^]_c_). The change of [Ca^2+^]_c_ is sensed by Miro1, resulting in mitochondrial fragmentation, Δ*Ψ*m loss, ATP reduction, and ROS burst. Thereafter, the damaged mitochondria induce apoptosis in a Bak‐dependent manner. In the presence of apoptotic inhibitor, the cell death type shifts to necroptosis.

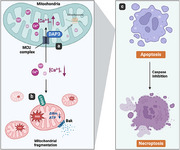

## 1 Introduction

In addition to ATP production, mitochondria participate in various cellular processes, such as generating reactive oxygen species (ROS), buffering Ca^2+^, and initiating apoptosis [1, 2]. Maintaining normal mitochondrial function is therefore essential for cell homeostasis and survival [3]. A unique feature of mitochondria is the presence of their own genome and translation machinery, known as mitoribosomes [4, 5]. Although the primary function of mitoribosomes is protein synthesis, recent studies have uncovered their novel roles in regulating apoptosis, cell metabolism, and RNA editing [6, 7, 8, 9, 10, 11, 12].

Death‐associated protein 3 (DAP3) is a subunit of the mitoribosome [13, 14, 15, 16, 17]. It was first identified by Kissil et al. in 1995 [19] as a mediator of interferon‐γ (IFN‐γ)‐induced apoptosis [18]. Subsequently, DAP3 was shown to mediate the apoptotic effects of several other cytokines, including tumor necrosis factor‐α (TNF‐α), Fas ligand (Fas‐L), and TNF‐related apoptosis inducing ligand (TRAIL) [19, 20]. Additionally, DAP3 contributes to anoikis, a type of cell death that occurs upon cell detachment from the extracellular matrix [21]. The expression of DAP3 is transcriptionally regulated by human endogenous retroviruses‐K (HML‐10) elements and microRNA‐365‐1 (miRNA‐365‐1) at the promoter region [22, 23]. Treating cells with IFN‐γ and chemotherapeutic drug cyclophosphamide (CTX) can significantly upregulate DAP3 levels through the HML‐10 and miRNA‐365‐1, respectively [22, 23]. The upregulation of DAP3 can be sufficient to induce apoptosis [22, 23], whereas downregulation of DAP3 has a protective effect against various apoptotic stimuli [18, 19, 21, 24], suggesting that DAP3 is a potent mediator of cell death. However, the reported mechanisms by which DAP3 mediates apoptosis remain controversial.

Depending on the origin of stimulation, apoptosis is classified into two distinct forms: extrinsic apoptosis and intrinsic apoptosis [25]. The extrinsic pathway is triggered by the binding of plasma membrane receptors with the extracellular ligands such as IFN‐γ, TNF‐α, and TRAIL. This interaction initiates the assembly of a signalling complex on the inner cell membrane to transmit the cell death motion [26]. In contrast, the intrinsic pathway is driven by irreversible and broad mitochondrial damage [27, 28]. Miyazaki and Reed [20] suggested that DAP3 acts as an adaptor, linking extrinsic cell death receptors (DRs) and Fas‐associated death domain (FADD) at the inner side of cell membrane. The formation of DR/DAP3/FADD complex consequently activates caspase‐8 and induces apoptosis [20]. A similar mechanism has been reported in DAP3‐induced anoikis [21]. However, evidence from other studies shows that DAP3 is retained inside mitochondria during apoptosis [29, 30, 31, 32]. In line with this, cotransfection of dominant‐negative FADD or caspase‐8 failed to prevent the cell death caused by DAP3 overexpression [19]. These findings indicate that additional mechanisms exist during the DAP3‐mediated cell death. Mitochondrial morphology change, especially excessive fragmentation, is a hallmark of apoptosis [33]. We and others have previously demonstrated that the expression level of DAP3 is critical to the maintenance of a healthy mitochondrial network [29, 34, 35]. Yet, little is known about how DAP3 affects mitochondrial health and whether the mechanism is related to its apoptotic function.

Here, we provide novel insights into the role of mitoribosomal protein DAP3 in the regulation of cell death. We found that DAP3 interacts with the mitochondrial calcium uniporter (MCU), leading to increased mitochondrial Ca^2+^ levels and decreased cytosolic Ca^2+^ levels. Consequently, this imbalance is sensed by the Rho GTPase Miro1, which induces excessive mitochondrial fragmentation, loss of mitochondrial membrane potential (Δ*Ψ*m), ATP decline, and oxidative stress. The damaged mitochondria trigger cell death via the intrinsic apoptotic pathway in a Bak‐dependent manner. Depletion of Miro1 or MCU alleviates mitochondrial fragmentation, ROS stress, and cell death. Our study reveals the functions of mitoribosomal protein DAP3 beyond its role in protein translation.

## 2 Results

### 2.1 Overexpression of DAP3 Induces Cell Death and Mitochondrial Damage

**FIGURE 1:**
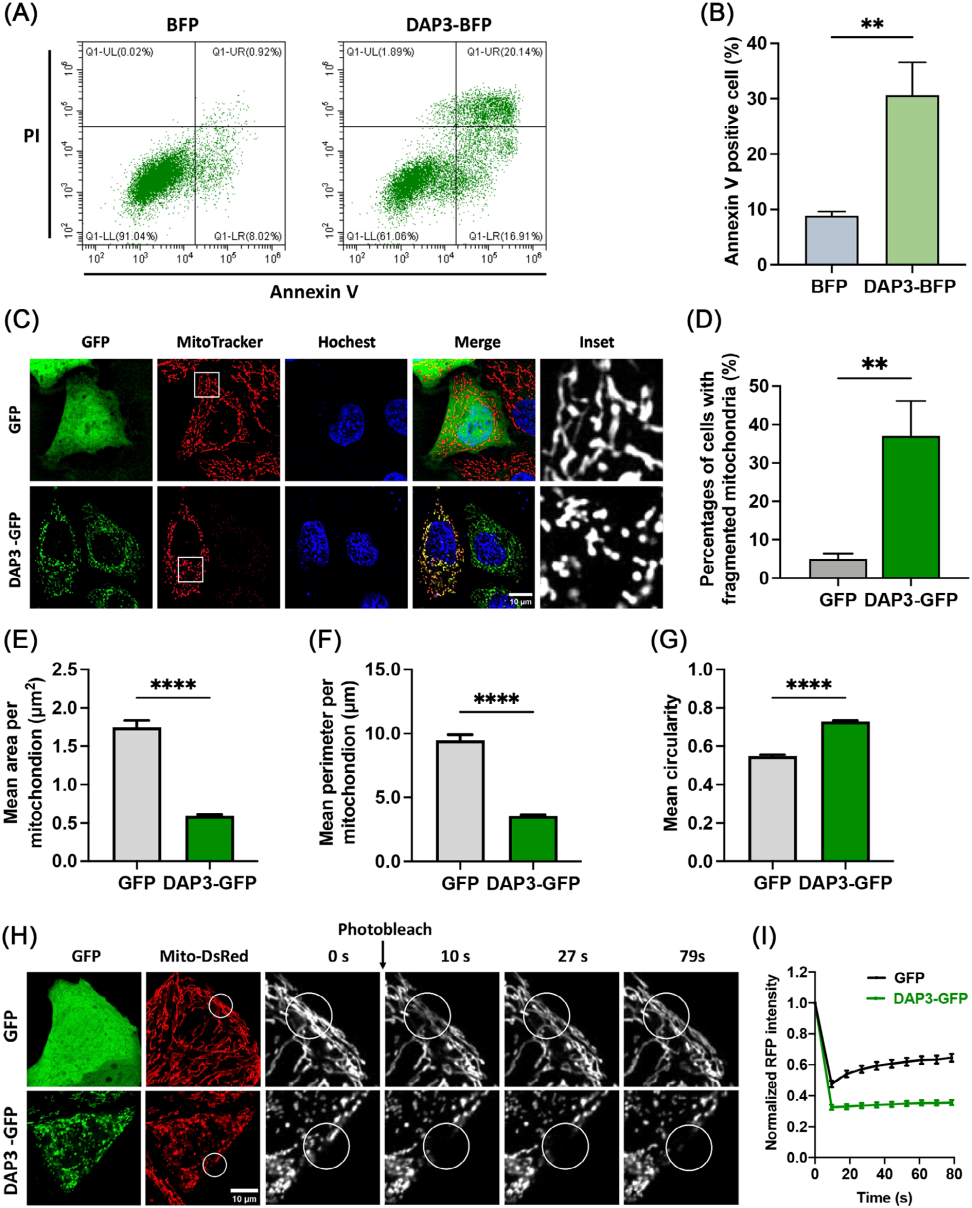
DAP3 induces cell death and mitochondrial fragmentation. (A) FACS assay of cell death in HeLa cells following transfection with vector–blue fluorescent protein (BFP) and DAP3–BFP for 24 h. The cells were stained with FITC‐Annexin V/PI. (B) Quantitative analyses of apoptotic cells (Annexin V positive) in (A). Data are mean ± SD from three independent experiments. (C) HeLa cells were transfected with vector–green fluorescent protein (GFP) and DAP3–GFP for 24 h, followed by staining with MitoTracker (red) and Hoechst (blue) to visualize mitochondria and nuclei, respectively. Representative images of live cells are shown. (D) Quantification of cells with fragmented mitochondria in (C). Data are mean ± SD (*n* > 300 cells from three independent experiments). (E–G) Quantification of mitochondria size (mean area, perimeter) and shape (circularity) per mitochondrion using ImageJ software (Particle analysis). Data are mean ± SEM (*n* = 3207 and 5825 mitochondria from twenty GFP and DAP3–GFP transfected cells, respectively, in three independent experiments). (H) HeLa cells stably expressing mitochondria‐targeted DsRed (mito‐DsRed) were transfected with vector–GFP and DAP3–GFP for 24 h, followed by FRAP analysis. A circular region of interest (ROI) was placed on the mitochondria and photobleached with a 561 nm laser. (I) Normalized curves of fluorescence recovery in (H). Scale bar, 10 µm. *p < 0.05, **p < 0.01, ***p < 0.001, ****p < 0.0001.

Upregulation of DAP3 levels has been reported to induce cell death [22, 23]. However, the underlying mechanism remains poorly understood and controversial results have been observed in previous studies [18–23, 29–32]. To investigate the roles of DAP3 in cell death, we first overexpressed C‐terminal BFP‐tagged DAP3 in HeLa cells. After 24 h, DAP3–BFP overexpression induced cell death in approximately 30.7 ± 5.9% of the transfected cells. In contrast, the percentage of cell death observed in cells transfected with the control BFP vector was low at approximately 8.9 ± 0.8% (Figure 1A,B).

We and others have previously revealed that DAP3 is a mitoribosomal subunit involved in the regulation of mitochondrial dynamics [13, 29, 34]. Since mitochondrial fragmentation is a typical feature of apoptosis, we investigated the impact of DAP3 overexpression on mitochondrial morphology and dynamics. Mitochondria were visualized by staining with MitoTracker dye. As shown in Figure 1C,D, DAP3 disrupted the balance of mitochondrial dynamics, resulting in extensive mitochondrial fragmentation in approximately 37.0 ± 9.2% of the cells, in contrast to only about 5.0 ± 1.4 3% in the control group, which is consistent with the previous report [29]. Quantitative analyses of the mitochondrial average size revealed a decrease in mitochondrial mean area from 1.75 ± 0.01 to 0.59 ± 0.01 µm^2^ (Figure 1E), and a reduction in mean perimeter from 9.47 ± 0.44 to 3.56 ± 0.07 µm (Figure 1F). On the other hand, the mean circularity increased from 0.55 ± 0.01 to 0.73 ± 0.01 (Figure 1G), confirming a shift in mitochondrial morphology from tubular‐like to dot‐like. To further validate the mitochondrial fragmentation phenotype, we conducted the fluorescence recovery after photobleaching (FRAP) assay to measure mitochondrial interconnectivity. As shown in Figure 1H,I, the mitochondria in control cells (GFP‐overexpressed) exhibited rapid fluorescence recovery after photo‐bleaching within the initial 30 s, whereas this recovery process was markedly impeded upon DAP3–GFP overexpression, resulting in sustained darkness in the bleached region. These results further support the notion that DAP3 serves as a potent inducer of mitochondrial fragmentation.

**FIGURE 2:**
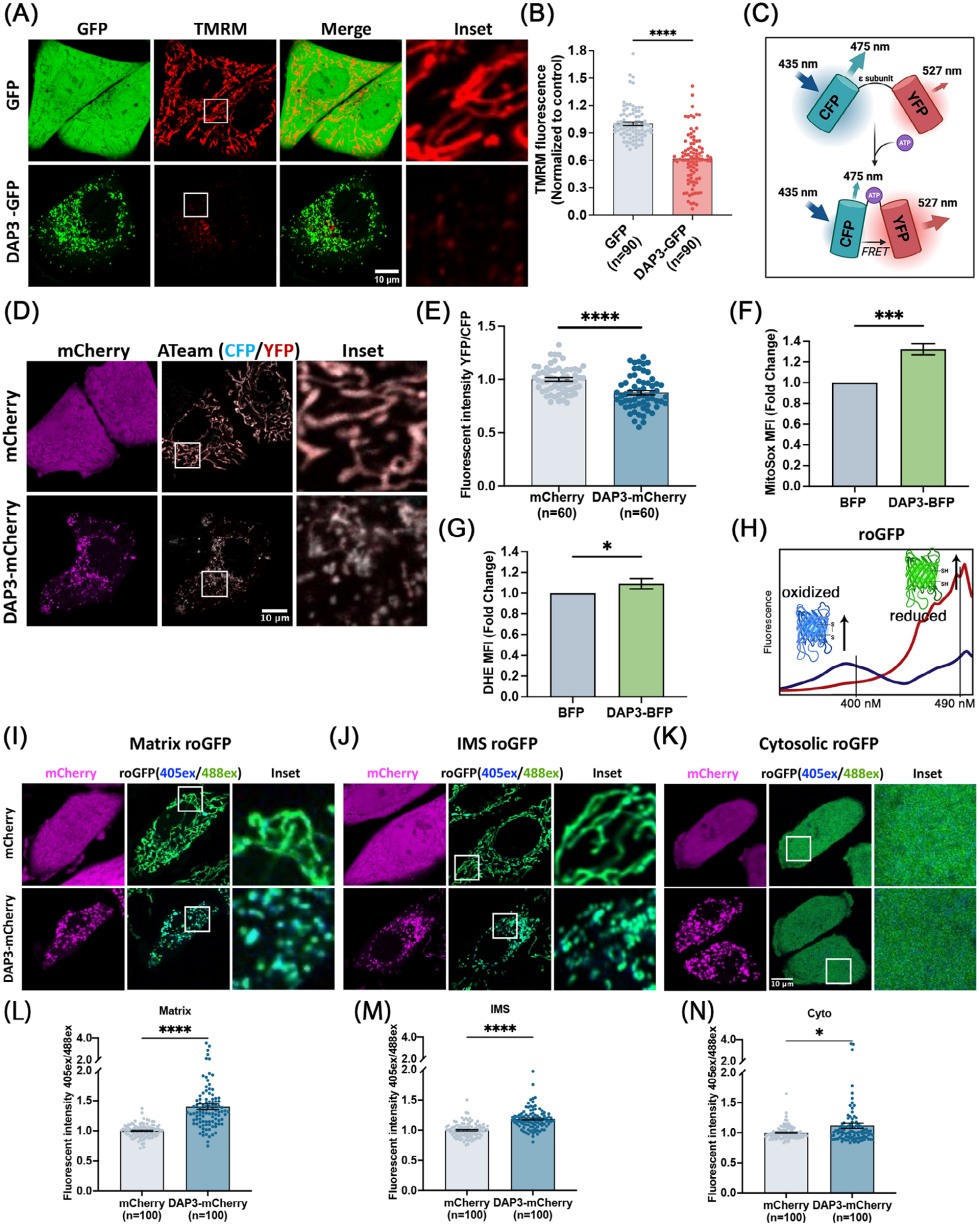
DAP3 triggers Δ*Ψ*m loss and ROS increase. (A) HeLa cells were transfected with vector–GFP and DAP3–GFP for 24 h, followed by staining with tetramethylrhodamine methyl ester (TMRM) for Δ*Ψ*m measurement. Representative images of live cells are shown. (B) Quantification of TMRM fluorescent intensity in (A). Data are mean ± SEM (*n* = 90 cells from three independent experiments). (C) Schematic drawing of the ATeam probe. The probe consists of a CFP and a YFP connected by the ε subunit of the F_0_F_1_–ATP synthase. In ATP‐free state, the *ε* subunit adopts an extended and flexible conformation, keeping the two fluorescent proteins apart. Upon ATP binding, the *ε* subunit brings the two fluorescent proteins into proximity, which enhances FRET efficiency and increases YFP emission. (D) HeLa cells were cotransfected with vector–GFP/DAP3–GFP and mitochondrial matrix‐localized ATeam probe for 24 h, followed by confocal microscopy. Representative images of live cells are shown. (E) Quantification of ATeam fluorescent in (D). Data are mean ± SEM (*n* = 60 cells from three independent experiments). (F and G) HeLa cells were transfected with vector–BFP and DAP3–BFP. After 24 h, cells were harvested and stained with MitoSOX or DHE, followed by FACS analysis to determine mitochondrial and cytosolic ROS, respectively. The fold changes of mean fluorescence intensity (MFI) are shown in the bar chart. (H) Excitation peaks of redox‐sensitive GFP (roGFP) probe. Upon oxidization of the surface cysteines, the excitation peak of roGFP shifts from ∼490 nm (red curve) to ∼400 nm (blue curve). The fluorescence intensities excited at ∼490 and ∼400 nm can be used as a ratiometric indicator of the redox state of cells. (I–K) HeLa cells stably expressing mitochondrial matrix‐localized (I), IMS‐localized (J), and cytosol‐localized (K) roGFP were transfected with vector–mCherry or DAP3–mCherry. Fluorescent images of roGFP were collected. The oxidized and reduced forms of roGFP were displayed with blue and green colors, respectively. (L–N) Quantitative analyses of the roGFP emission ratio in (I–K). Data are mean ± SEM (*n* = 100 cells from three independent experiments). Scale bar, 10 µm. *p < 0.05, **p < 0.01, ***p < 0.001, ****p < 0.0001.

Notably, weaker MitoTracker signals, indicating a loss of Δ*Ψ*m, were observed in some of the DAP3‐overexpressing cells (Figure 1C). To confirm this observation, we performed TMRM staining for quantitative analysis of the Δ*Ψ*m. Overexpression of DAP3 resulted in a significant decline in mean TMRM intensity by approximately 38.1% in the fragmented mitochondria (Figure 2A,B), implying Δ*Ψ*m collapse and mitochondrial damage. In addition to membrane potential, we evaluated the mitochondrial ATP levels using a fluorescence resonance energy transfer (FRET)‐based indicator, ATeam (Figure 2C) [36, 37]. ATeam is designed by linking a CFP tag and a YFP tag with the ε subunit of F_0_F_1_–ATP synthase. Upon ATP binding, the *ε* subunit brings the two fluorescent proteins close to each other, thus enhancing YFP emission via FRET. In line with the reduced Δ*Ψ*m, DAP3 overexpression also impaired mitochondrial ATP production (Figure 2D,E).

Given that DAP3 is a subunit of mitoribosome, we assessed whether its overexpression perturbs mitochondrial protein synthesis. As shown in Figure S1A, the levels of mitochondrial gene‐encoded proteins, ND5 and COXII, remained unimpaired, suggesting DAP3 regulates cell death and mitochondrial homeostasis through a mechanism independent of its role in protein translation. To ascertain whether DAP3‐induced mitochondrial fragmentation is a common phenomenon in other cell types, we overexpressed DAP3–GFP in HEK 293T, U2OS, and ARPE‐19 cells. Consistently, mitochondrial fragmentation, accompanied by reduced mitochondrial size, was observed in all these cell lines (Figure S1B,C).

A burst of ROS has been widely recognized as a trigger of mitochondrial damage and cell death [38]. To extend the observations on mitochondrial health, intracellular ROS levels were measured using mitochondrial matrix‐ and cytosolic‐specific dyes, respectively. As expected, overexpression of DAP3 elevated ROS levels in both mitochondrial matrix and cytosol (Figure 2F,G). The average ROS levels increased by approximately 30% in the matrix, while the rise in the cytosol was comparatively lower by 10%, but still significant.

To further examine ROS levels in different subcellular localizations, three types of redox‐sensitive GFP (roGFP) probes were stably expressed in HeLa cells: Matrix‐roGFP (localizes to mitochondrial matrix) [39], IMS‐roGFP (specifically localizes to the mitochondrial intermembrane space) [40], and Cyto‐roGFP (localizes in the cytosol) [39]. roGFP contains two surface‐exposed cysteines, enabling the reversible formation of an intramolecular disulfide bridge, thereby acting as a sensor of redox status. roGFP exhibits two excitation peaks at ∼ 400 and ∼490 nm, and one emission peak at ∼510 nm. Oxidation of roGFP leads to the increase of the 400 nm excitation peak, while reduction enhances the 490 nm excitation peak, providing a ratiometric method for the detection of ROS (Figure 2H) [41]. Overexpression of DAP3–mCherry in roGFP stable cell lines led to a significant increase in ROS levels across all the measured subcellular localizations (Figure 2I–K). Particularly, the mitochondrial matrix exhibited the highest increase in ROS (1.4‐fold) (Figure 2I,L), followed by the IMS (1.2‐fold) (Figure 2J,M) and cytosol (1.1‐fold) (Figure 2K,N), indicating that the ROS may originate from the mitochondrial matrix and subsequently spread into the IMS and cytosol after mitochondrial membrane damage or via diffusion.

Overall, the above results demonstrate that DAP3 can lead to mitochondrial fragmentation, loss of Δ*Ψ*m, ATP decline, and oxidative stress, indicating that DAP3 potentially triggers cell death via the intrinsic pathway.

### 2.2 Overexpression of DAP3 Induces Cell Death via Intrinsic Pathway

**FIGURE 3:**
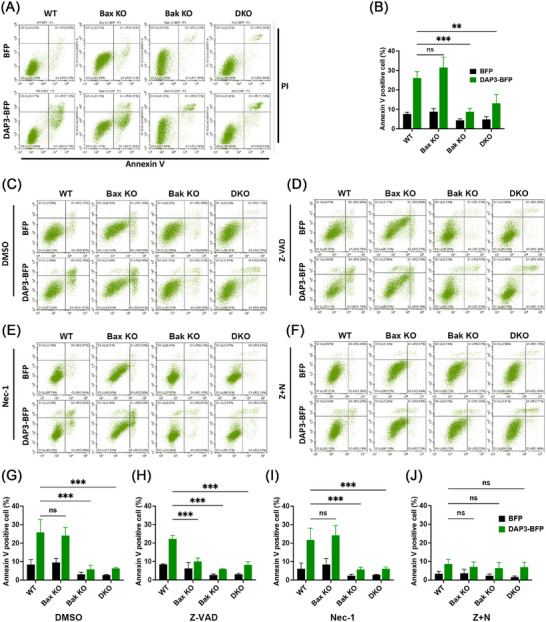
DAP3 regulates cell death via intrinsic pathway. (A) FACS assay of cell death in WT, Bax KO, Bak KO, and Bax/Bak DKO MEF cells following transfection of vector–BFP and DAP3–BFP for 24 h. The cells were stained with FITC‐Annexin V/PI. (B) Quantitative analyses of apoptotic cells (Annexin V positive) in (A). Data are mean ± SD from three independent experiments. (C–F) FACS assay of cell death in WT, Bax KO, Bak KO, and DKO MEF cells following transfection with vector–BFP and DAP3–BFP for 24 h in the presence of DMSO, Z‐VAD (Apoptotic inhibitor, 80 µM), necrostatin‐1 (necroptotic inhibitor, 80 µM), or Z‐VAD + necrostatin‐1 (80 + 80 µM). (G–J) Quantitative analyses of apoptotic cells (Annexin V positive) in (C–F). Data are mean ± SD from three independent experiments.

It is well established that mitochondrial outer membrane permeabilization, regulated by Bax and Bak, is a key event of intrinsic cell death [42]. To investigate the involvement of DAP3 in mediating the intrinsic cell death, we took advantage of using Bax/Bak knockout (KO) MEF cell lines. Approximately 24 h after transfection with DAP3–BFP or vector–BFP, the cells were harvested and stained with propidium iodide (PI)/Annexin V for cell death measurement. As shown in Figures 3A,B, and S2A, the deletion of both Bak and Bax (double KO, DKO) reduced cell death from approximately 26.1 ± 3.3 to 13.1 ± 4.5%. Interestingly, while Bak KO effectively inhibited the cell death rate, Bax KO had protective effect, with approximately 31.5 ± 5.4% of cells undergoing cell death after DAP3 overexpression. These findings reveal that DAP3 induces cell death via the intrinsic pathway in a Bak‐dependent manner.

To further clarify that the observed cell death is apoptosis rather than another form of cell death, the apoptotic inhibitor Z‐VAD‐FMK (Z‐VAD) was added to the cell culture. Surprisingly, the addition of Z‐VAD failed to inhibit the cell death in WT cells overexpressing DAP3, with a cell death rate of 22.2 ± 1.8% in Z‐VAD‐treated cells (Figure 3D,H) compared with 25.8 ± 6.9% in the DMSO‐treated control (Figure 3C,G). This phenomenon is reminiscent of necroptosis, a form of programmed necrotic cell death that can be triggered when apoptosis is blocked. To confirm this, the necroptosis blocker necrostatin‐1 (Nec‐1) was added into the cell culture, either alone (Figure 3E,I) or in combination with Z‐VAD (Figure 3F,J). The combination of Nec‐1 and Z‐VAD significantly blocked cell death after DAP3 overexpression, lowering the percentage of apoptotic cells to 8.66 ± 2.4% (Figure 3F,J). In contrast, Nec‐1 alone did not prevent the cell death (21.7 ± 6.3%) (Figure 3E,I). Therefore, these results indicate that DAP3 primarily induces intrinsic apoptosis, while in the presence of an apoptotic inhibitor, the mode of cell death changes to necroptosis.

An interesting observation is that although Z‐VAD alone failed to prevent cell death in WT cells, it showed protective effects in Bax KO cells, reducing the cell death to 10.0 ± 1.9% (Figure 3D,H). Thus, the combination of Z‐VAD treatment and Bax depletion had a similar effect as the combination of Z‐VAD and Nec‐1, suggesting that Bax may play a role in regulating necroptosis.

The Bcl‐2 family proteins are known to regulate cell death by direct interaction with Bax and Bak. To examine whether DAP3 induces cell death via a similar mechanism, we performed a coimmunoprecipitation (co‐IP) assay. However, no detectable interaction between DAP3 and Bax/Bak was observed, as shown in Figure S2B,C. To further investigate the DAP3's effects, we examined mitochondrial morphology in Bak KO cells after DAP3 overexpression. The results revealed that while Bak deletion was sufficient to block cell death, it did not prevent mitochondrial fragmentation (Figure S2D). Likewise, the ROS accumulation was not alleviated in Bak KO cells (Figure S2E,F). These data suggest that Bak may act as a downstream effector in DAP3‐induced mitochondrial fragmentation and ROS burst.

### 2.3 DAP3 Induces Mitochondrial Impairment Independently of the Canonical Fusion–Fission Machinery

**FIGURE 4:**
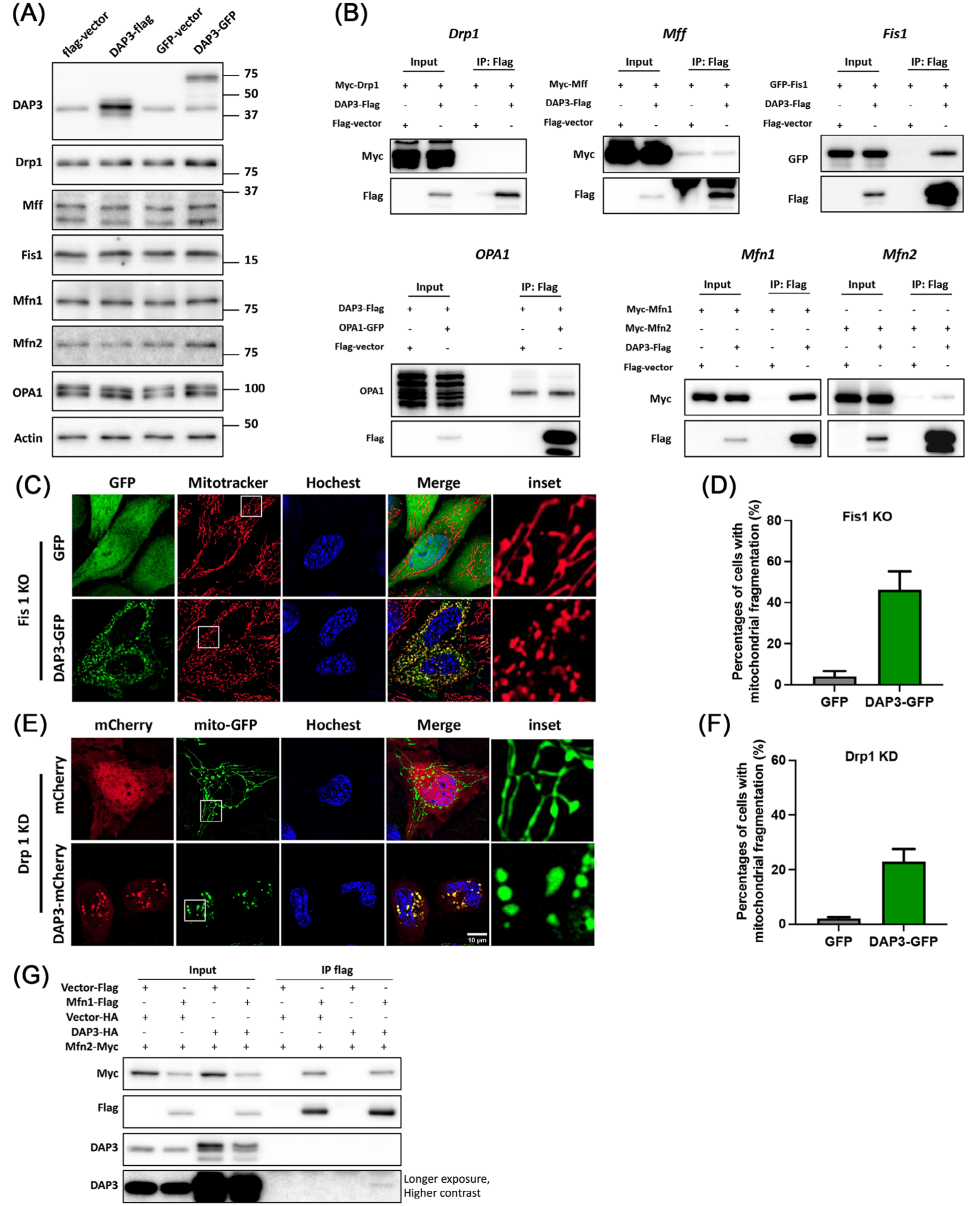
DAP3 induces mitochondrial fragmentation independent of canonical fusion/fission machinery. (A) HeLa cells were transfected with vector–flag/GFP and DAP3–flag/GFP. After 24 h, cells were harvested, and the levels of mitochondrial fusion/fission proteins were analyzed by western blot. (B) DAP3 interacts with Fis1, Mfn1, and Mfn2. HEK293 cells cotransfected with DAP3–flag and indicated plasmid of fusion/fission proteins were collected after 24 h for coimmunoprecipitation (IP) with anti‐Flag beads, followed by western blot analysis. (C) Fis1 knockout (KO) HeLa cells were transfected with vector–GFP and DAP3–GFP for 24 h. Mitochondrial morphology was visualized with MitoTracker. (E) HeLa cells stably expressing mito‐GFP were transfected with Drp1 SiRNA for 48 h, followed by overexpression of vector–mCherry or DAP3–mCherry for another 24 h. Representative images of live cells are shown. (D and F) Quantification of cells with fragmented mitochondria in (C and E). Data are mean ± SD (*n* > 300 cells from three independent experiments). (G) HEK293 cells were cotransfected with the Mfn1–flag and Mfn2–myc with or without DAP3–flag for 24 h. Cell lysates were used for co‐IP with anti‐flag beads, followed by western blot. Scale bar, 10 µm.

Mitochondrial dynamics, regulated by fusion and fission proteins such as Mfn1/2, OPA1, Drp1, Fis1, and Mff, plays a crucial role in controlling mitochondrial morphology. Enhanced mitochondrial fragmentation can arise from either increased fission or decreased fusion. To elucidate how DAP3 triggers mitochondrial fragmentation, we examined whether DAP3 overexpression affects the levels of the fusion–fission proteins. As shown in Figure 4A, the protein levels of these mitochondrial dynamics mediators remained unchanged following DAP3 overexpression. To further explore the mechanism, we performed a co‐IP assay to investigate potential associations between DAP3 and these fusion–fission proteins. Interestingly, DAP3 was found to interact with Fis1 and Mfns (Figure 4B).

To determine whether the fission mediator Fis1 is responsible for the DAP3‐induced mitochondrial fragmentation, we utilized a Fis1 KO cell line (Figure S3A). Overexpression of DAP3 in Fis1 KO cells still resulted in substantial mitochondrial fragmentation (46.23 ± 9.05%; Figure 4C,D), thus excluding the involvement of Fis1. Although Drp1 showed no interaction with DAP3 (Figure 4B), it is widely characterized as a key regulator of mitochondrial fission. We, therefore, investigated its potential involvement. Depletion of Drp1 using siRNA significantly inhibited mitochondrial fission, leading to a highly fused mitochondrial network (Figures S3A and 4E; GFP control). However, overexpression of DAP3–GFP still induced mitochondrial fission in approximately 22.9 ± 4.6% of the Drp1 knockdown (KD) cells (Figure 4F), albeit with a slight decrease compared with that in WT cells (37.0 ± 9.2%) (Figure 1D). These results suggest that Fis1 and Drp1 are largely dispensable for DAP3‐induced mitochondrial fragmentation.

Mfns mediate mitochondrial fusion by forming homodimers or heterodimers that bring two mitochondria together. Given the observed interaction between DAP3 and Mfns, it is conceivable that DAP3 might disrupt Mfns’ dimer formation, thereby promoting mitochondrial fragmentation. To explore this hypothesis, we carried out co‐IP assays to examine whether DAP3 interferes with Mfns interaction. As shown in Figure 4G, overexpression of DAP3 did not reduce the quantity of Mfn2 pulled down by Mfn1. Besides, comparing the precipitated protein levels with the input protein levels revealed that the Mfn1–DAP3 interaction was notably weaker than that of Mfn1–Mfn2. To further validate that DAP3 does not disrupt the interaction between Mfns, we performed a co‐IP assay of Mfns in the presence of a substantial quantity of purified recombinant DAP3 (Figure S3B,C). Notably, the addition of 200 µg of DAP3 protein to the cell lysate (containing 400 µg of total protein) did not impair the Mfn1–Mfn2 interaction. We also tried to rescue the mitochondrial fragmentation by overexpressing Mfn1. However, Mfn1 overexpression resulted in a clustered, grape‐like mitochondrial network that was distinct from the original tubular structure (Figure S3D), consistent with previous reports [43, 44, 45]. Consequently, it remains challenging to ascertain whether Mfn1 overexpression effectively rescues DAP3‐induced mitochondrial fragmentation.

Although DAP3 is a mitochondrial ribosomal protein, previous research has associated it with the extrinsic cell death pathway, which takes place in the cytosol [20, 21]. Additionally, very recent studies have identified novel functions of DAP3 in RNA editing and splicing, revealing that a portion of DAP3 localizes in the nucleus [7, 46]. This raises a pivotal question regarding the subcellular localization of DAP3 during its role in mitochondrial fragmentation. To address this, we constructed an N‐terminal GFP‐tagged DAP3 (GFP–DAP3). The existence of GFP obstructs the recognition of the N‐terminal mitochondrial targeting sequence, resulting in the retention of GFP–DAP3 in the cytosol. Remarkably, cells expressing GFP–DAP3 did not exhibit mitochondrial fragmentation (Figure S3E), suggesting that DAP3's mitochondrial localization is indispensable for its function.

In summary, these data suggest that DAP3 overexpression induces mitochondrial fragmentation through a mechanism that operates independently of the canonical fusion–fission mechanisms.

### 2.4 Miro1 Mediates the DAP3‐Induced Mitochondrial Impairment

**FIGURE 5:**
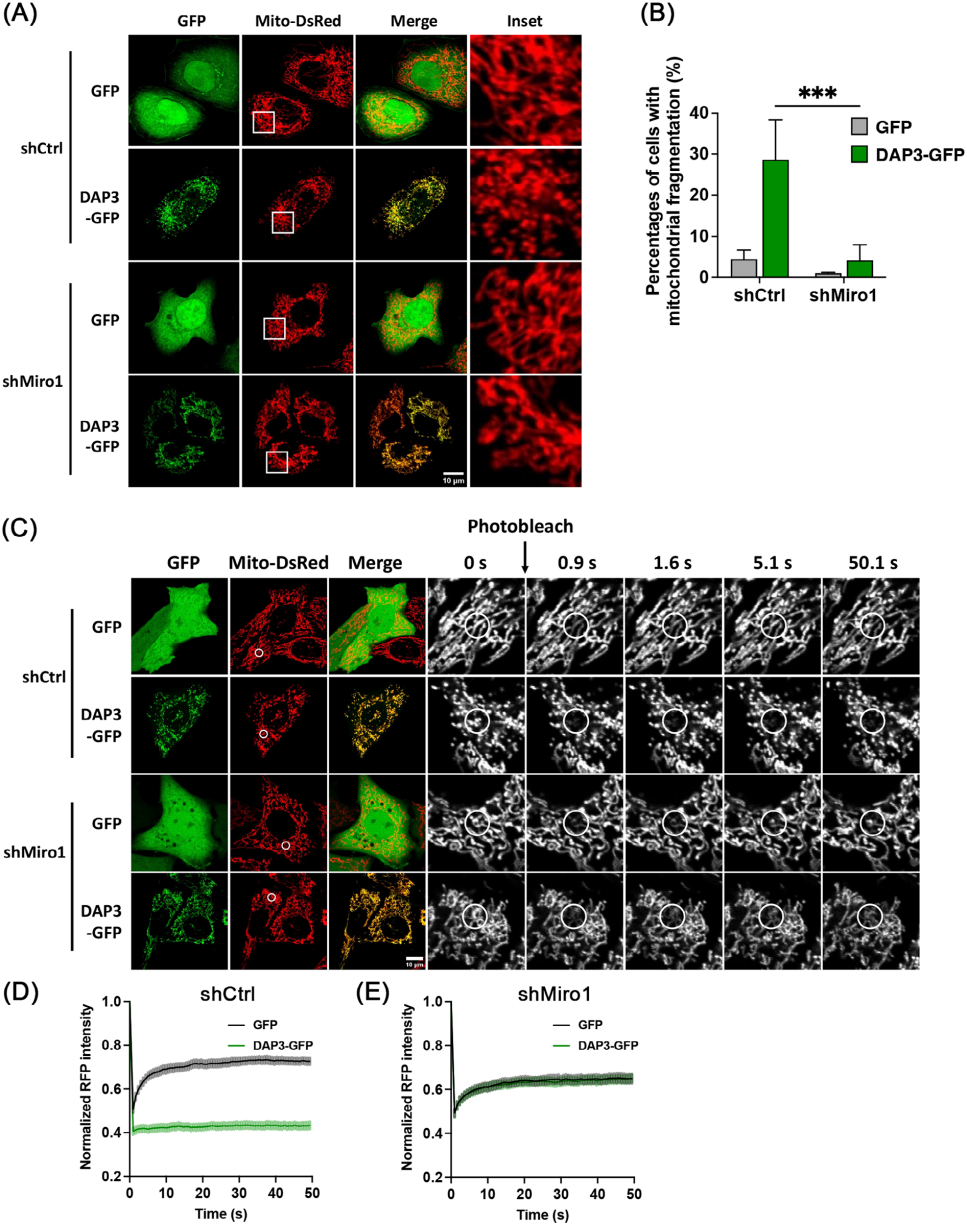
DAP3 induces mitochondrial fragmentation via Miro1. (A) HeLa cells stably expressing indicated shRNA and mito‐DsRed were transfected with vector–GFP and DAP3–GFP for After 24 h. Representative images of live cells are shown. (B) Quantification of cells with fragmented mitochondria in (A). Data are mean ± SD (*n* > 300 cells from three independent experiments). (C) HeLa cells stably expressing indicated shRNA and mito‐DsRed were transfected with vector–GFP and DAP3–GFP for 24 h, followed by FRAP assay. A circular ROI was placed on the mitochondria and photobleached with a 561 nm laser. (D ansd E) Normalized curves of FRAP assay in (C). Data are mean ± SEM (*n* = 30 cells from three independent experiments). Scale bar, 10 µm.

Miro1, a mitochondrial outer membrane protein, regulates mitochondrial movement by forming a transporting complex with kinesin heavy chain isoform 5 (KIF5) and trafficking kinesin‐binding protein 1 and 2 (TRAK1/2) [47]. Recent research has identified Miro1 as a mediator of mitochondrial fragmentation independent of the canonical fusion–fission machinery [48, 49]. Therefore, we sought to investigate the involvement of Miro1 in DAP3‐induced mitochondrial fragmentation. To this end, we generated stable KD HeLa cell lines using lentiviral short hairpin RNA (shRNA) constructs targeting either Miro1 gene or LacZ as a control. We then overexpressed DAP3–GFP in these cell lines. As shown in Figures 5A,B and S4A, Miro1 KD significantly reduced the percentage of cells with fragmented mitochondria to 4.1 ± 3.8%, compared with 28.7 ± 9.7% in shCtrl cells. The FRAP assay showed similar recovery curves after DAP3–GFP and vector–GFP overexpression in shMiro1 cell lines, further confirming that Miro1 is responsible for the DAP3‐induced mitochondrial fragmentation (Figure 5C–E).

**FIGURE 6:**
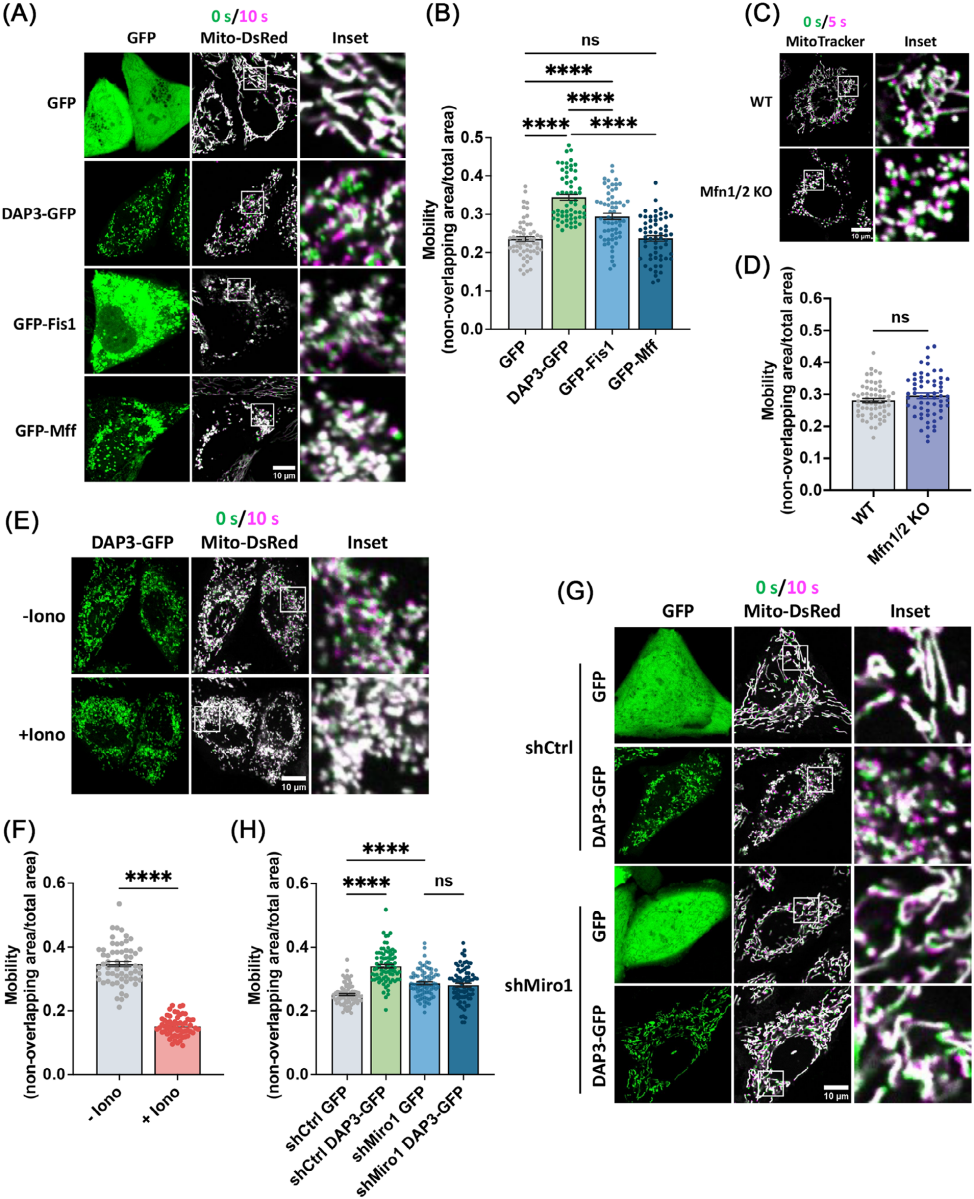
DAP3 promotes mitochondrial movement. (A) HeLa cells stably expressing mito‐DsRed were transfected with vector–GFP, DAP3–GFP, GFP–Fis1, and GFP–Mff for 24 h. Two images with a 10‐s interval were obtained for each cell, and the mitochondria were colored green and magenta, respectively. The two mitochondria images were subsequently overlaid. Representative images of live cells are shown. (B) Quantitative analyses of mitochondrial motility in (A). Motility was represented by the percentage of nonoverlapping area in the total mitochondrial area. (C) Mitochondria in WT and Mfn1/2 KO MEF cells was visualized by MitoTracker staining. The live cell images were captured as in (A) with a 5‐s interval. (D) Quantitative analyses of mitochondrial motility in (C). (E) HeLa cells stably expressing mito‐DsRed were transfected with DAP3–GFP for 24 h. Thereafter, 5 µM of ionomycin was add to the cell culture (DMEM contains ∼1.8 mM Ca^2+^) to trigger a surge of intracellular calcium. The images were captured as in (A) immediately. (F) Quantitative analyses of mitochondrial motility in (E). (G) HeLa cells stably expressing indicated shRNA and mito‐DsRed were transfected with vector–GFP and DAP3–GFP for After 24 h. The images were captured as in (A). Representative images of live cells are shown. (H) Quantitative analyses of mitochondrial motility in (G). Scale bar, 10 µm. *p < 0.05, **p < 0.01, ***p < 0.001, ****p < 0.0001.

Given the pivotal role of Miro1 in mitochondrial movement, we next investigated whether the fragmentated mitochondria in DAP3‐overexpressing cells have altered mobility. To evaluate mitochondrial movement, we acquired two images at a 10‐s interval, labeling them green and magenta, respectively. The two images were subsequently overlaid, with increased mitochondrial mobility indicated by a greater nonoverlapping mitochondrial area [50]. The percentage of nonoverlapping areas was measured for quantitative comparison. As shown in Figure 6A,B, fragmented mitochondria in DAP3–GFP‐overexpressing cells displayed significantly more nonoverlapping areas (35.5 ± 0.5%) compared with controls (22.8 ± 0.9%), indicating increased mitochondrial mobility. These results support the notion that Miro1 takes effects to induce mitochondrial fragmentation in DAP3‐overexpressing cells. In contrast, overexpression of fission protein (Fis1 or Mff) or deletion of fusion proteins (Mfn1/2) resulted in extensive mitochondrial fragmentation but did not increase mitochondrial mobility as DAP3 did (Figures 6A–D and S4B), consistent with our previous conclusion that DAP3 induces mitochondrial fragmentation independently of the canonical fusion–fission machinery.

As a Ca^2+^‐binding protein, Miro1 regulates the mitochondrial transport in a Ca^2+^‐dependent manner [51]. High levels of intracellular calcium have been demonstrated to suppress Miro1‐mediated mitochondrial movements [52, 53]. To further substantiate that DAP3 promotes mitochondrial movements via Miro1, we triggered a surge in the intracellular Ca^2+^ levels by adding ionomycin, a calcium ionophore, to the cell culture. The addition of ionomycin immediately suppressed mitochondrial movement (Figure 6E,F). In line with this, KD of Miro1 also attenuated the increase in mitochondrial motility induced by DAP3 overexpression (Figure 6G,H). These results collectively confirm that Miro1 promotes mitochondrial movement in DAP3‐overexpressing cells.

**FIGURE 7:**
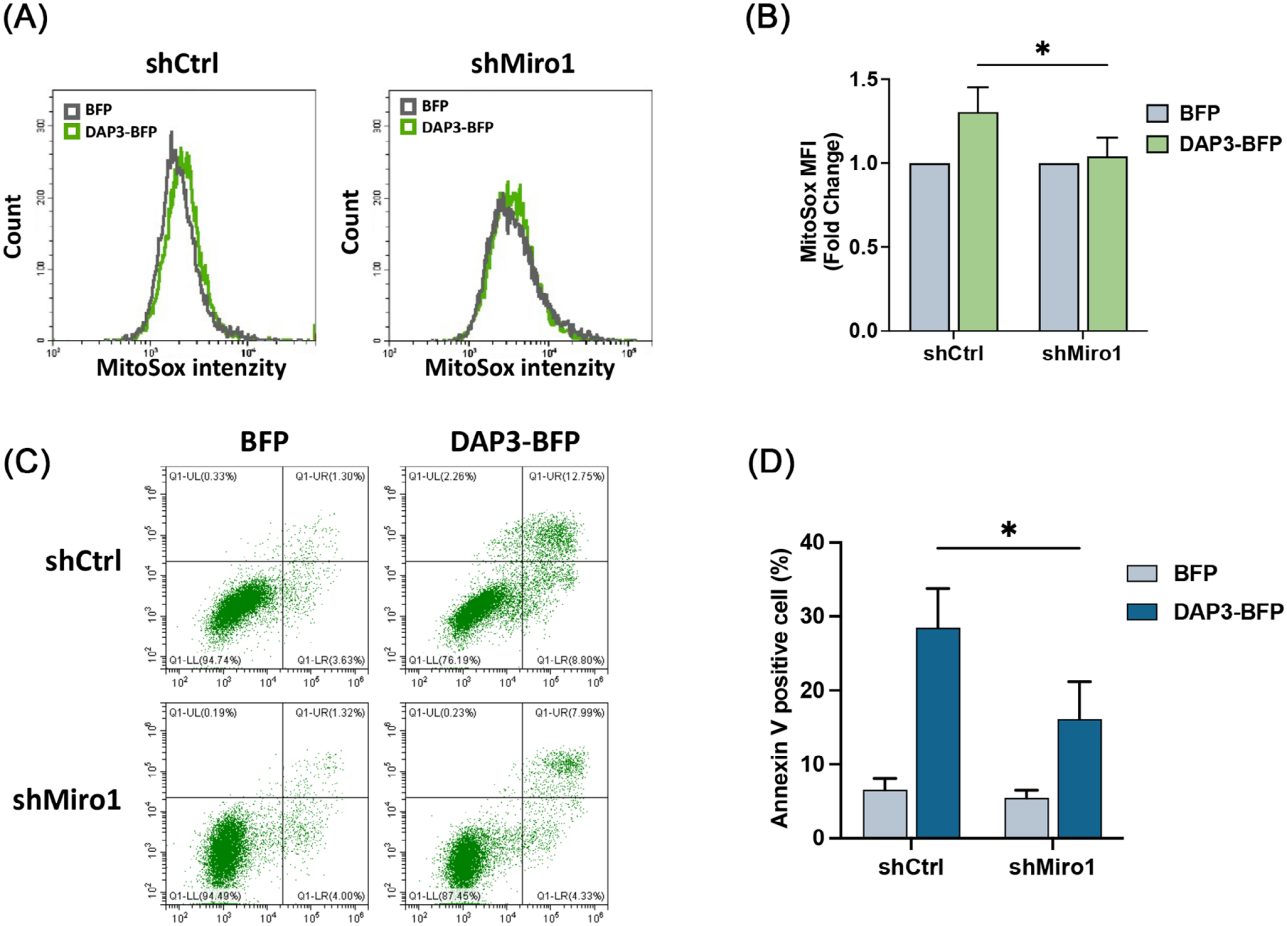
Miro1 KD alleviates DAP3–induced ROS burst and cell death. (A and B) HeLa cells stably expressing indicated shRNA were transfected with vector–BFP and DAP3–BFP. After 24 h, cells were harvested and stained with MitoSOX, followed by FACS analysis to determine mitochondrial ROS. The fold changes of mean fluorescence intensity are shown in the bar chart. Data are mean ± SD from three independent experiments. (C) FACS assay of cell death in shControl and shMiro1 HeLa cells following transfection with vector–BFP and DAP3–BFP for 24 h. The cells were harvested and stained with FITC‐Annexin V/ PI. Data are mean ± SD from three independent experiments. (D) Quantitative analyses of apoptotic cells (Annexin V positive) in (C). Data are mean ± SD from three independent experiments. *p < 0.05, **p < 0.01, ***p < 0.001, ****p < 0.0001.

Previous studies have demonstrated that mitochondrial fragmentation can lead to increased mitochondrial ROS generation [54, 55]. Therefore, we sought to investigate whether Miro1‐mediated mitochondrial fragmentation contributes to ROS generation in DAP3‐overexpressing cells. To assess mitochondrial ROS levels, we overexpressed DAP3–GFP and vector–GFP in shCtrl and shMiro1 cells, followed by MitoSox dye staining. As shown in Figures 7A,B and S4C, KD of Miro1 attenuated the increase of mitochondrial ROS, revealing that Miro1 is necessary for DAP3 to promote ROS generation. Moreover, as mitochondria‐derived ROS is one of the key factors for apoptosis [56, 57], we examined whether Miro1 KD could alleviate DAP3‐triggered cell death. shCtrl or shMiro1 HeLa cells were transfected with DAP3–BFP or vector–BFP, followed by a cell death assay. As shown in Figure 7C,D, shMiro1 cells exhibited a lower apoptosis rate (16.1 ± 5.1%) compared with shCtrl cells (28.5 ± 5.3%) after DAP3 overexpression, confirming that Miro1‐mediated mitochondrial fragmentation and the following ROS burst contribute to DAP3‐induced cell death.

### 2.5 DAP3 Regulates Intracellular Ca^2+^ Levels

**FIGURE 8:**
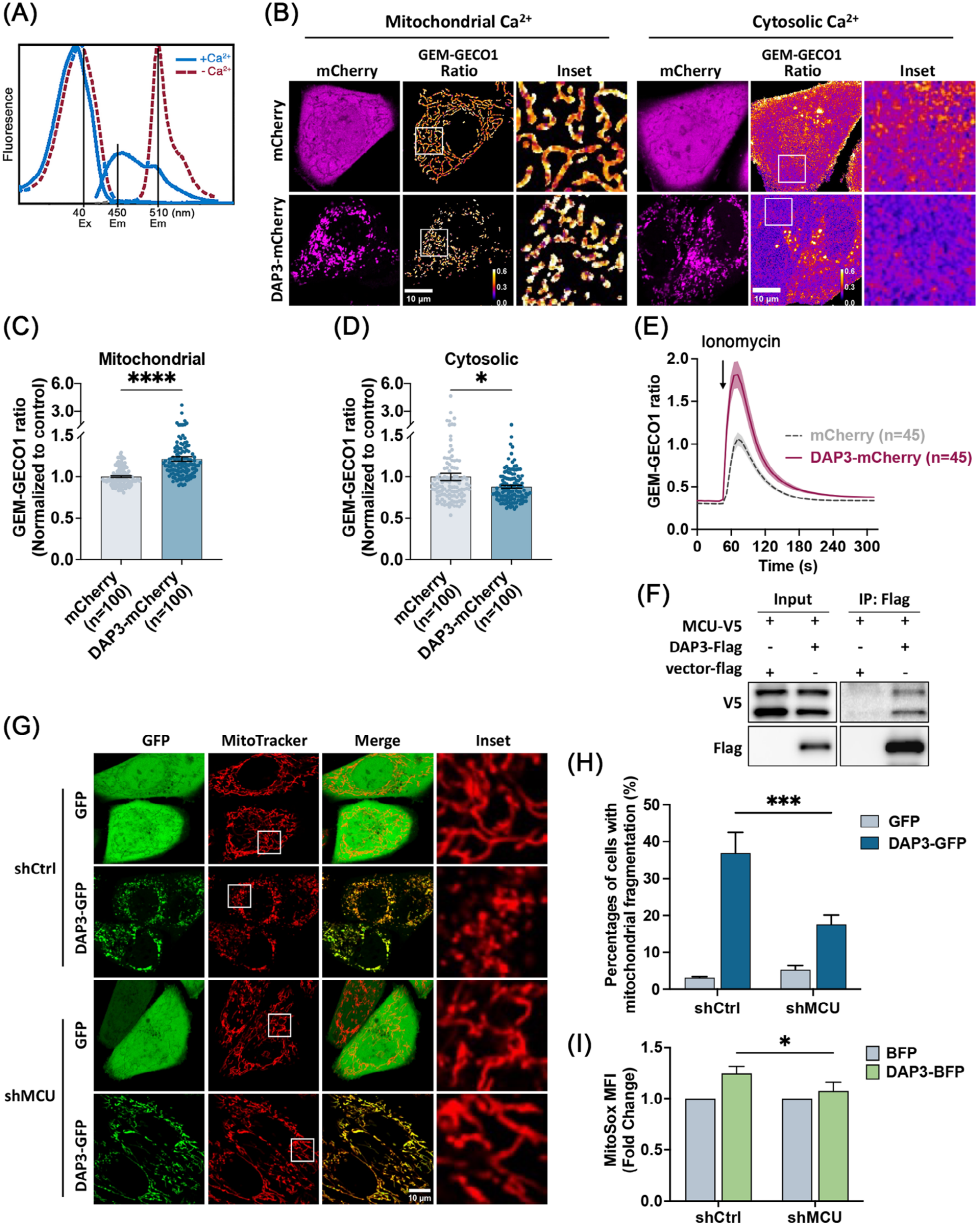
DAP3 alters intracellular calcium via MCU complex. (A) Excitation and emission peaks of calcium‐sensitive GFP (GEM–GECO1) probe. Upon Ca^2+^ binding, the emission peak of GEM–GECO1 shifts from ∼510 to ∼450 nm. The fluorescence intensities emitted at these two peaks can be used as a ratiometric indicator of the Ca^2+^ level. (B) Analysis of basal Ca^2+^ level. HeLa cells stably expressing mitochondria matrix‐localized (left panel) and cytosol‐localized (right panel) GEM–GECO1 were transfected with vector–mCherry or DAP3–mCherry. Fluorescent images of GEM–GECO1 were collected. The emission ratios of GEM–GECO1 (450–510 nm) were presented in spectrum. (C and D) Normalized GEM–GECO emission ration in (B). Data are mean ± SEM (*n* = 100 cells). (E) Curves of mitochondrial Ca^2+^ uptake. HeLa cells stably expressing mitochondria matrix‐localized GEM–GECO1 were transfected with vector–mCherry or DAP3–mCherry. After 24 h, the Ca^2+^ uptake was stimulated by ionomycin with the presence of 500 nM CaCl_2_. Time‐lapse curves of the GEM–GECO1 emission ratio are presented. Data are mean ± SEM (*n* = 45 cells from three independent experiments). (F) DAP3 interacts with MCU. HEK293 cells cotransfected with DAP3–flag and MCU‐V5 for 24 h were collected for coimmunoprecipitation (IP) with anti‐Flag beads, followed by western blot analysis. (G) HeLa cells stably expressing indicated shRNA were transfected with vector–GFP and DAP3–GFP for 24, followed by staining with MitoTracker. Representative images of live cells are shown. (H) Quantification of cells with fragmented mitochondria in (F). Data are mean ± SD (*n* > 300 cells from three independent experiments). (I) HeLa cells stably expressing indicated shRNA were transfected with vector–BFP and DAP3–BFP. After 24 h, cells were harvested and stained with MitoSOX, followed by FACS analysis to determine mitochondrial ROS. The fold changes of mean fluorescence intensity are shown. Data are mean ± SD from three independent experiments. Scale bar, 10 µm. *p < 0.05, **p < 0.01, ***p < 0.001, ****p < 0.0001.

Miro1 acts as a Ca^2+^ sensor that regulates mitochondrial transport [51]. Perturbations of cytosolic Ca^2+^ levels ([Ca^2+^]_c_) have been reported to cause Miro1‐mediated mitochondrial fragmentation [48]. To examine whether DAP3 overexpression affects intracellular Ca^2+^ levels, the [Ca^2+^]_c_ and mitochondrial Ca^2+^ level ([Ca^2+^]_m_) were evaluated using the ratiometric probe GEM–GECO1 [58], which specifically localizes to the cytosol and mitochondria matrix. Ca^2+^ binding to the GEM–GECO1 probe causes a shift in emission wavelength from ∼510 toward ∼450 nm (Figure 8A), thus allowing detection of changes in Ca^2+^ levels by comparing the emission ratio at the two wavelengths. As depicted in Figure 8B–D, DAP3–mCherry overexpression led to increased [Ca^2+^]_m_ and decreased [Ca^2+^]_c_ in cells with fragmented mitochondria, indicating that DAP3 can induce alterations in basal Ca^2+^ levels.

We next investigated whether DAP3 accelerates mitochondrial Ca^2+^ uptake, thereby leading to the observed changes in basal Ca^2+^ levels. Mitochondrial Ca^2+^ uptake was triggered by adding ionomycin in the presence of 500 nM CaCl_2_. As shown in Figure 8E, DAP3–mCherry overexpression markedly enhanced mitochondrial Ca^2+^ uptake, suggesting that DAP3 regulates intracellular Ca^2+^ homeostasis by promoting mitochondrial Ca^2+^ uptake. Interestingly, a recovery of mitochondrial morphology was observed during the measurement (Figure S5A), supporting our earlier finding that DAP3 induces mitochondrial fragmentation via altering intracellular Ca^2+^ levels.

To delve further into the mechanism by which DAP3 regulates intracellular Ca^2+^, we searched the BioGRID protein interaction database. Interestingly, a recent study on mitochondrial interaction networks revealed a potential interaction between DAP3 and the MCU, a subunit of the calcium channel in the inner mitochondrial membrane that controls the mitochondrial Ca^2+^ influx [59]. To verify the interaction, we coexpressed Flag‐tagged DAP3 and V5‐tagged MCU in HEK 293T cells, followed by co‐IP with anti‐Flag beads. As demonstrated in Figure 8F, DAP3 successfully pulled down MCU, suggesting that DAP3 physically associates with MCU. We then constructed a stable HeLa cell line with MCU KD using shRNA (Figure S5B). In this cell line, mitochondrial fragmentation was significantly reduced (17.6 ± 2.5%) compared with the control (36.9 ± 5.6%; Figure 8G,H). Furthermore, KD of MCU attenuated the DAP3‐induced increase in mitochondrial ROS (Figure 8I). Together, these findings indicate that the MCU channel is essential for DAP3‐induced changes in basal Ca^2+^ levels and mitochondrial stress.

## 3 Discussion

Mitochondria originated from bacteria that entered a primitive host cell, a process known as endosymbiosis, around 2 billion years ago [60]. During evolution, most genes from the ancestral endosymbionts were either lost or transferred to the nuclear genome of host cell [61]. As a result, more than 99% of mitochondrial proteins are encoded by the nuclear DNA and synthesized in the cytosol. Only 13 proteins are produced inside mitochondria by the mitoribosome [62]. This raises the question of why this translational system and mitochondrial genome have been retained, given the very limited protein output. The colocation for redox regulation hypothesis suggests that the retention allows rapid control of gene expression in response to changes in mitochondrial redox state [63]. Additionally, Björkholm et al. [64] suggested that intramitochondrial translation is critical for the correct localization of highly hydrophobic membrane proteins. Another possibility is that mitoribosomes have critical functions beyond translation, making them necessary for mitochondria. In line with this, a few mitoribosomal subunits, including DAP3, small ribosomal subunit protein uS5m, large ribosomal subunit protein mL41, large ribosomal subunit protein bL33m, and large ribosomal subunit protein mL65/PDCD9, have been found to play roles in controlling apoptosis, cell metabolism, and RNA editing over the past two decades [6, 7, 8, 9, 10, 11, 12]. Such regulatory capacity could be critical for mitochondrial adaptation to various physiological or pathological conditions.

DAP3 was initially identified as a modulator of cell death through RNA interference screening. Silencing of the DAP3 gene conferred cells with resistance to IFN‐γ or TNF‐α caused cell death [18], while upregulation of DAP3 levels was sufficient to trigger cell death in several types of cells [19, 22, 23]. It was proposed that DAP3 mediates apoptosis by linking FADD to the TRAIL receptors DR4 and DR5 [20]. However, later evidence from independent research groups demonstrated that DAP3 is a mitoribosomal subunit [13, 29, 34], with its mitochondrial localization remaining unchanged during cell death, despite the observed release of cytochrome c [30, 31, 32]. These observations challenged the previously proposed pathways in which DAP3 was thought to function in the cytosol. Here, we present data revealing that DAP3 promotes cell death through the intrinsic pathway. Several lines of evidence support this notion. First, overexpression of DAP3 induces changes in mitochondrial morphology, resulting in excessive fragmentation (Figure 1C–I). Along with this, a significant reduction in Δ*Ψ*m and mitochondrial ATP levels was observed, implying a loss of mitochondrial integrity (Figure 2A–E). Second, a notable increase in ROS was detected following DAP3 overexpression (Figure 2F–N). Given the well‐established fact that mitochondrial ROS are potent triggers of mitochondrial damage and cell death [65], it is plausible that DAP3 stimulates cell death by inducing oxidative stress. This possibility was supported by our findings that KD of Miro1 alleviated ROS stress and cell death (Figure 7). Third, intrinsic apoptosis is executed by the Bcl‐2 family proteins, Bak and Bax [66]. Our results show that DAP3‐mediated cell death was significantly blocked by Bak KO or Bak/Bax double KO, indicating that DAP3 functions via the intrinsic pathway (Figure 3A,B).

Mitochondrial dysfunction has been reported as a trigger of necroptosis in several studies, which illustrate the roles of the mitochondrial permeability transition pore (MPTP), ROS, and other factors in promoting the formation of necrosome and the activation of downstream necroptotic pathways [67, 68, 69]. Interestingly, our investigations reveal a dual role for DAP3 in determining cell fate. While the primary role of DAP3 is to trigger apoptosis, the presence of a caspase inhibitor can alter DAP3's function, redirect it toward inducing necroptosis. Bax appears to be involved in this necroptotic process, supported by the observation that Z‐VAD treatment in a Bax KO cell line blocked cell death (Figure 3D,H). In a previous study, Bax was reported to play a role in Ca^2+^ overload‐induced necrotic cell death by promoting MPTP opening [70]. Of note, this function of Bax is independent of its apoptotic ability, as the oligomerization‐defective Bax(63‐65A) can mediate MPTP opening in Bax/Bak DKO cells [70]. Our observation is consistent with this study, showing that Bax is not involved in DAP3‐induced apoptosis but plays a role in the regulation of necroptosis.

Our findings reveal that DAP3 induces mitochondrial fragmentation via Miro1 rather than the canonical fusion–fission machinery (Figures 4 and 5). Miro1 is a component of the transporting complex that localizes on the mitochondrial outer membrane [53, 71, 72]. Cytosolic calcium negatively regulates mitochondrial motility by binding to the EF‐hand domain of Miro1 [52]. Here, we provide evidence showing that DAP3 regulates resting intracellular calcium levels. Overexpression of DAP3 leads to increased [Ca^2+^]_m_ and decreased [Ca^2+^]_c_. Consequently, Miro1 can sense the decrease in [Ca^2+^]_c_, allowing for increased mitochondrial transporting activity. The precise mechanism by which Miro1 mediates mitochondrial fragmentation remains unclear. It is plausible that Miro1 promotes fission by generating the mechanical forces required for mitochondrial separation [73].

The MCU complex is the Ca^2+^‐specific channel in the inner mitochondrial membrane [74, 75]. Our results demonstrate that DAP3 interacts with MCU and promotes mitochondrial Ca^2+^ uptake (Figure 8E,F). Moreover, MCU KD attenuated the DAP3 overexpression‐induced mitochondrial fragmentation and ROS accumulation, indicating that DAP3 mediates intracellular Ca^2+^ levels through the MCU complex (Figure 8G–I). Further investigations are needed to elucidate how DAP3 regulates the MCU complex. Several pieces of evidence suggest that the GTP‐binding domains in DAP3 are critical for its apoptotic function [19, 20, 22, 29]. Therefore, it would be valuable to explore whether these GTP binding domains structurally and functionally regulate the DAP3–MCU interaction in future studies.

We previously demonstrated that DAP3 KD also triggers mitochondrial fragmentation by reducing the phosphorylation of Drp1 at Ser‐637 [34]. Mechanistically, the dephosphorylation of Ser‐637 is controlled by the Ca^2+^ dependent phosphatase calcineurin [34, 76]. According to this study, DAP3 depletion could lead to an increase in [Ca^2+^]_c_, which in turn activates calcineurin. The activated calcineurin can dephosphorylate Drp1 at Ser‐637, thus promoting mitochondrial fission [76]. The role of DAP3 in regulating Ca^2+^ reconciles the mitochondrial fragmentation phenotype induced by both overexpression and depletion of DAP3, suggesting that a finely tuned level of DAP3 is critical for mitochondrial homeostasis.

Mitochondria rely heavily on the nuclear‐encoded proteins for their function, necessitating significant intercompartmental crosstalk with the nucleus. This interconnectedness enables cells to adapt to the ever‐changing cellular conditions [77]. As a nuclear‐encoded protein, the expression level of DAP3 is influenced by several pathophysiological conditions [22, 23, 78, 79]. For example, IFN‐γ, typically produced during immune responses to enhance the anti‐microbial activity of immune cells [80], is known to upregulate DAP3 expression [22]. In line with this, when we activated mouse bone marrow‐derived macrophages (BMDMs) with inflammatory stimuli (IFN‐γ and LPS), a notable increase in DAP3 levels was observed (Figure S6), suggesting that DAP3 may play a potential role in macrophage activation. It is well established that proinflammatory macrophages rely mainly on glycolysis instead of oxidative phosphorylation [81]. Thus, DAP3 is unlikely to contribute to macrophage activation by facilitating the synthesis of mitochondrial respiratory chain proteins. Here, we demonstrate that the upregulation of DAP3 induces the production of mtROS, a double‐edged sword. While excessive ROS can lead to cell death, accumulating evidence suggests that increased mtROS is essential for the activation of immune responses [82]. Therefore, it is tempting to speculate that DAP3 may facilitate the functional adaptation of immune cells, such as macrophages, by promoting ROS generation.

The present study has some limitations. First, this study did not comprehensively characterize mitochondrial metabolism following DAP3 overexpression, which could potentially provide deeper insights into unknown functions of DAP3. Second, although our results suggest a link between DAP3 and immune responses, further experimental validation is required to confirm this association. Finally, we focused on the in vitro model to explore the molecular functions of DAP3 in the present study. Additional in vivo research under physiological or pathological conditions will be essential to explore the significance of DAP3.

**FIGURE 9:**
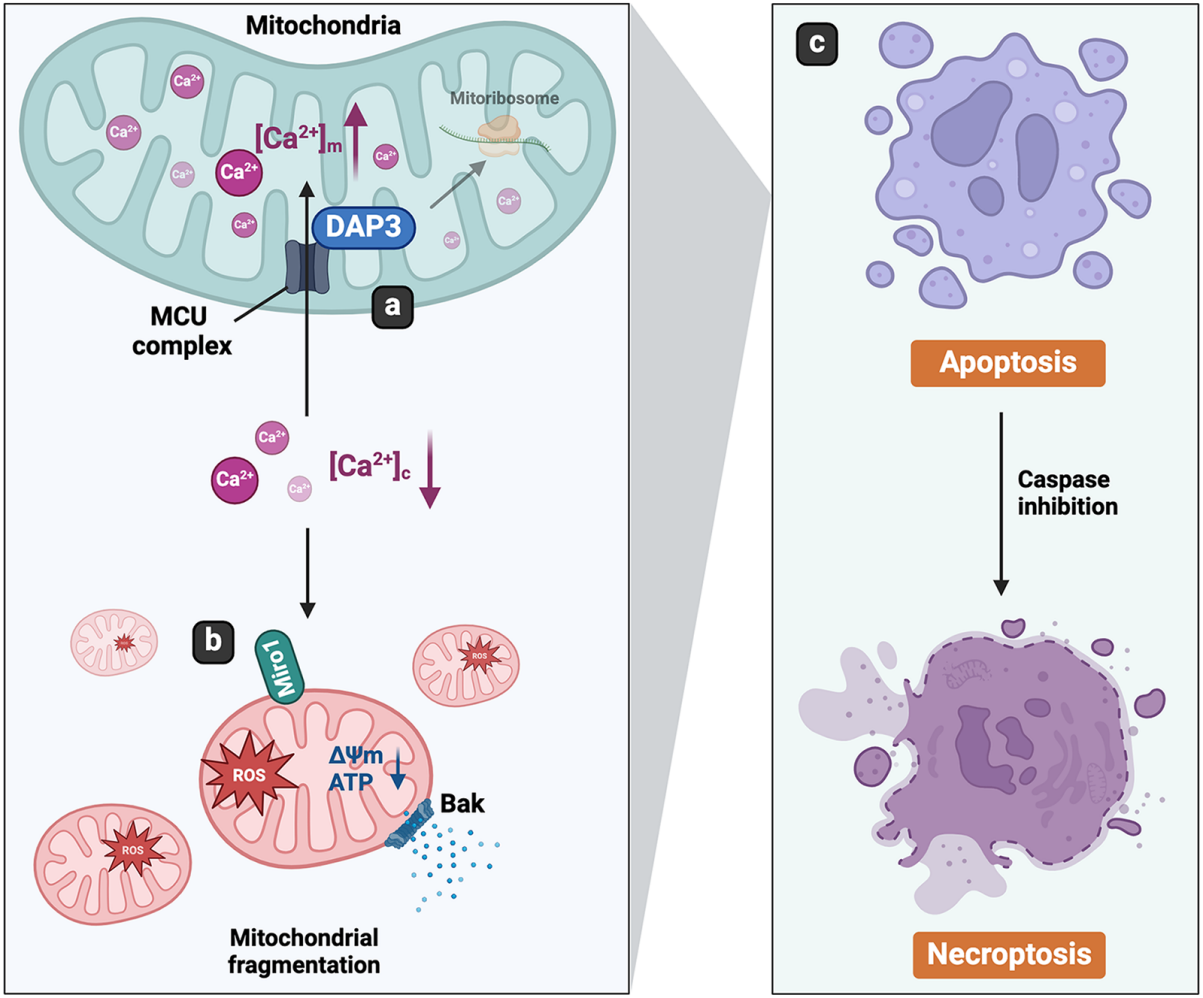
Schematic diagram. (A) DAP3 regulates intracellular Ca^2+^ via the MCU complex. Upregulated level of DAP3 promotes mitochondrial calcium uptake, leading to the decrease of [Ca^2+^]_c_. (B) Thereafter, the change of [Ca^2+^]_c_ is sensed by Miro1, resulting in mitochondrial fragmentation, Δ*Ψ*m loss, ATP decline, and ROS burst. (C) The damaged mitochondria induce apoptosis in a Bak‐dependent manner. In the presence of apoptotic inhibitor, the cell death type shifts to necroptosis.

In summary, this study uncovers a novel role of mitoribosomal protein DAP3 in regulating intracellular calcium levels, thereby controlling mitochondrial homeostasis and cell fate. DAP3 promotes mitochondrial calcium accumulation via the MCU complex, leading to a decrease in [Ca^2+^]_c_. This change in [Ca^2+^]_c_ triggers Miro1‐mediated mitochondrial damage, including excessive fragmentation, loss of Δ*Ψ*m, and ROS overproduction. Ultimately, the damaged mitochondria induce Bak‐dependent intrinsic apoptosis (Figure 9).

## 4 Materials and Methods

### 4.1 Flow Cytometry Analysis

For the ROS assay, cells were harvested and washed once with PBS. Mitochondrial and cytosolic ROS were measured by incubating live cells in cell imaging buffer (140 mM NaCl, 2.5 mM KCl, 1.8 mM CaCl_2_, 1.0 mM MgCl_2_, 20 mM HEPES, pH 7.4) supplemented with 5 µM MitoSox or DHE, respectively, at 37°C for 20 min. After the incubation period, cells were washed twice and resuspended in imaging buffer for flow cytometry assay (Beckman; Cytoflex LX). For cell death assay, all cells, including floating and attached, were collected and washed once with PBS. Subsequently, they were incubated in binding buffer supplemented with Annexin V/propidium iodide (PI) (BioLegend; 640906). About 10,000 events were recorded for each experiment and the results were analyzed using CyExpert software. Cells showing negative staining were regarded as living cells, whereas Annexin V staining indicated apoptosis. Cells showing positive staining for both Annexin V and PI were classified as late apoptotic or necrotic cells.

### 4.2 Fluorescence and Confocal Microscopy Imaging

Fluorescence and confocal microscopy imaging were conducted following previously established protocols [83]. Cells were seeded onto a glass‐bottom dish and transfected with 1.5 µg of plasmid. Live cells adhered to the dish were observed and imaged using the Olympus FV3000 confocal microscopy system or the Olympus IX83 microscopy system with a chamber to maintain a temperature of 37°C and 5% CO_2_. ImageJ/Fiji software was used for quantitative analyses of the mitochondrial size, number, and fluorescence. Mitochondrial segmentation was performed using the Mitochondria Analyzer plugin in ImageJ [84], and morphological parameters were measured by particle analysis with the segmented mitochondria. The levels of ROS, mitochondrial potential, and calcium levels were calculated by quantifying the fluorescence values of mitochondrial or cytosolic probes per cell.

MitoTracker Red CMXRos staining was performed at a concentration of 100 nM for 20 min. TMTM staining was performed at a concentration of 50 nM for 20 min. MitoSox and DHE staining were performed at concentration of 5 µM for 20 min. Hoechst 33342 staining of nuclei was performed at a concentration of 1 µg/mL in combination with other dyes.

The basal level of Ca^2+^ was measured in the cell culture medium at 37°C. GEM–GECO1 was excited at 405 nm with emission filter settings as approximately 450 and 510 nm. For imaging of ionomycin‐induced mitochondrial Ca^2+^ influx, cells were washed twice and incubated with 1 mL HEPES (20 mM)‐buffered Hanks balanced salt solution (HHBSS, Mg^2+^‐free) containing 500 nM CaCl_2_. Time‐lapse images were collected with 6.5 s intervals for 4 min. Approximately 45 s after the initial recording, 1 mL HHBSS containing 500 nM CaCl_2_ and 5 µM ionomycin was added to the dish using a dropper. The changes in mitochondrial and cytosolic Ca^2+^ levels were monitored by the fluorescence of GEM–GECO1.

For the measurement of mitochondrial ATP levels, mitochondria‐localized ATeam1.03‐nD/nA probe was excited at 405 nm, and emissions around 475 and 527 nm were scanned.

### 4.3 FRAP‐Based Mitochondrial Fusion Assay

A circular region of interest (ROI) was selected and photobleached by a single‐pulse 488 nm laser. Time‐lapse images were recorded with 0.94 s intervals. Ten cells were analyzed in each replicate. The fluorescence intensity of mito‐RFP in the ROIs at each time point was recorded and normalized to the initial fluorescence.

### 4.4 co‐IP and Western Blot

co‐IP experiments were performed as previously described. Briefly, HEK 293T cells were transfected with the target plasmids in 10 cm dishes. After 24 h, the cells were washed and harvested in 1 mL mammalian lysis buffer (50 mM Tris–HCl, 10% glycerol, 1% Triton X‐100, 100 mM NaCl, and 0.5 mM MgCl_2_, pH 7.4) supplemented with protease inhibitors, including 10 µg/mL of aprotinin, 1 mM phenylmethylsulfonyl fluoride (PMSF), 1 µM pepstatin, and 10 µM leupeptin, followed by centrifugation under 21,130×*g* for 20 min at 4°C. The supernatant was incubated with 5 µL M2 anti‐flag beads (Sigma) for 2 h at 4°C. After incubation, the beads were washed three times and suspended in 50 µL 2× SDS loading buffer for 10 min at 95°C to denature the precipitated proteins.

For the Western blot assay, protein samples were separated on 10–15% SDS‐PAGE and transferred to PVDF membranes. The membranes were blocked with 5% (W/V) skimmed milk in TBST buffer (20 mM Tris, 150 mM NaCl, 0.1% Tween20, pH 7.4). Incubation with primary antibodies was performed at 4°C overnight, followed by incubation with secondary antibodies (Santa Cruz) at room temperature for 1–2 h. The membranes were imaged using a gel‐doc Amersham Imager 600 system after brief incubation with ECL substrate.

### 4.5 Protein Expression and Purification Using *E. coli* System

To purify DAP3 for the co‐IP assay, the pET42b (+) plasmid harboring the DAP3 sequence was transformed into BL21 *E. coli*. Bacterial cultures were shaken at 200 rpm and 37°C until the OD_600_ value reached 0.6. DAP3 expression was initiated after the addition of 0.4 mM IPTG, followed by further cultivation at 180 rpm and 18°C overnight. Bacteria were then harvested by centrifugation (5000×*g* for 10 min), and resuspended in lysis buffer (0.4% Sarkosyl, 25 mM Tris–HCl, 500 mM NaCl, 10 mM β‐mercaptoethanol, 1% Tween‐20, pH 7.0) containing 1 mM PMSF, lysed by sonication, and centrifuged at 20,000×*g* for 1 h at 4°C. The supernatant containing DAP3 protein was purified by HisTrap HP column (Cytiva), HiTrap SP HP column (Cytiva), and Superdex 75 gel filtration column (Cytiva) according to the previous study [85]. The protein concentration was measured using the Bio‐Rad Protein Assay Kit (#5000006).

### 4.6 Statistical Analysis

Statistical analysis was conducted using GraphPad Prism software. Student's *t*‐test was used for comparing means between two groups, while one‐way ANOVA was used for comparing means among three or more groups. For assays with two independent variables, two‐way ANOVA was used. A *p* value < 0.05 was considered statistically significant

## Author Contributions

D. H. and Y‐C. L. conceptualized and designed the study. D. H. developed of methodology. D. H. and Y‐C. L. wrote, reviewed, and revised the paper. D. H. provided acquisition, analysis and interpretation of data, and statistical analysis. Q. Y., H. X., H. Z., and M. K. B. provided technical and material support. M. W., H. Z., V. C. Y., and M. K. B. provided insightful discussions. All authors read and approved the final manuscript.

## Ethics Statement

All experimental designs and protocols involving animals were approved by the Laboratory Animal Welfare and Ethics Committee of Army Medical University of China (no. AMUWEC20234511).

## Conflicts of Interest

Author Yih‐Cherng Liou is an Editorial board member of MedComm. Author Yih‐Cherng Liou was not involved in the journal's review of or decisions related to this manuscript. The other authors declared no conflict of interest.

## Supporting information



Supporting Information

## Data Availability

All raw data generated for this study are available from the corresponding author upon reasonable request.
