# Supplementary material for: Death‐Associated Protein 3 Triggers Intrinsic Apoptosis via Miro1 Upon Inducing Intracellular Calcium Changes
Source: MedComm (2020). 2025 May 10;6(5):e70214. doi: 10.1002/mco2.70214 (PMC12064944; doi:10.1002/mco2.70214)
Supplement: Supplementary file 1 — Supporting Information [file MCO2-6-e70214-s001.docx]

Supplementary Materials for

Mitochondrial ribosomal protein DAP3 triggers intrinsic apoptosis via Miro1 upon inducing intracellular calcium changes

Dongxue Hu^1^, Qiaoyun Yang^1^, Hongxu Xian^1,2^, Minghao Wang^3^, Hong Zheng^4^, Mallilankaraman Karthik Babu^5^, Victor C Yu ^6,7,8^, Yih-Cherng Liou^1,9*^

**This PDF file includes: Supplementary methods and Figure S1-6**

**Supplementary methods**

**Antibodies and reagents**

Antibodies: Actin (Santa Cruz, sc8432), Bak (Cell Signaling, 12105), Bax (Santa Cruz, sc-493), COXII (Abcam, ab79393), DAP3 (BD BioScience, 610662), Drp1 (BD BioScience, 611112), Fis1 (GeneTex, GTX111010), Flag-tag (Sigma-Aldrich, F3165), GAPDH (Santa Cruz sc32233), GFP (Santa Cruz sc-8334), HA-tag (Santa Cruz, sc7392), Hsp60 (Santa Cruz, sc-1052), MCU (Cell Signaling, 14997), Mff (Abcam, 139026), Mfn1 (Santa Cruz, sc-50330), Mfn2 (Santa Cruz, sc-100560), Miro1 (Santa Cruz, sc-398520), Myc-tag (Santa Cruz sc-789), ND5 (Abcam, ab92624), OPA1 (BD BioScience, 612606), Tubulin (Sigma-Aldrich, T5168), Tom20 (Santa Cruz, sc-17764).

Reagents: MitoTracker Red CMXRos (InvitrogenTM, M7512). Tetramethylrhodamine, methyl ester (TMRM; InvitrogenTM, T668). MitoSOXTM Red (InvitrogenTM, M36008), Dihydroethidium DHE (MedChemExpress, HY-D0079), Cell death assay kit (BioLegend, 640906), Z-vad-FMK (MedChemExpress, HY-16658B), Necrostatin-1 (MedChemExpress, HY-15760), Ionomycin (MedChemExpress, HY-13434)

**Plasmid construction**

All plasmids used in this project were constructed via either T4 ligase (NEB) or GibsonTM assembly according to the manufacturer’s protocol. Target genes were amplified by PCR and purified using the GeneJET Gel Extraction Kit (Thermo Scientific, K0692). Vectors and purified PCR products were digested with double restriction enzymes, followed by purification as described above. About 50 ng linearized vector and 100 ng target gene were used for T4 ligation or Gibson assembly, respectively. The reaction conditions were overnight at 4 °C for T4 ligase and 2 h at 50 °C for Gibson assembly, respectively. After ligation/Gibson assembly, the products were transformed into DH5α competent E. coli. For the pLV plasmid, Stable 3 competent strains were used to achieve better plasmid production. Transformed strains were spread and grown on LB agar plates supplemented with antibiotics for overnight. Single colonies were picked and cultured for plasmid extraction. After DNA sequencing, strains containing the correct construct were cultured for middle-scale plasmid extraction.

**Cell culture and transfection**

HeLa, HEK 293T, ARPE-19, U2OS, MEF, and generated KD/KO cells were cultured in Dulbecco’s modified Eagle’s medium (DMEM) (Hyclone), supplemented with 10% fetal bovine serum (FBS) (Gibco) and 10U/ml penicillin-streptomycin (Hyclone) at 37°C with 5% CO2. Transient transfections were carried out using Lipofectamine 2000 transfection reagent (Invitrogen) according to the manufacturer’s protocol. Cells were seeded one day before transfection to reach a density of 50-70% density for use the next day. Transfected cells were analysed 24 h post-transfection. The amount of plasmid used for each construct in a 35 mm glass bottom dish, 6-well plate, and 10 cm dish was 1.5 μg, 2 μg, and 10 μg, respectively.

**Culture and stimulation of BMDMs**

Bone marrow was extracted from the femurs and tibias of mice, and cultured in RPMI1460 (Hyclone) supplemented with 10% FBS and 10 ng/mL GM-CSF for 5 days to induce differentiation into bone marrow-derived macrophages (BMDMs). On day 6, IFN-γ (20 ng/mL) and LPS (10 ng/mL) were added to stimulate pro-inflammatory macrophages. The stimulation was performed for 2 days.

**RNA interference by siRNA**

RNA interference by siRNA was conducted using Lipofectamine 2000 according to the manufacturer’s protocol. Cells were seeded one day before transfection to reach a density of 50-70%. Twenty-four hours after siRNA treatment, cells were sub-cultured into a glass bottom dish to lower the cell density and then incubated for another 24 h. Thereafter, plasmids were transfected to overexpress the target proteins. Cells were harvested for further investigations at 72 h post-treatment. Scramble siRNA was used as a control. The siRNA sequences used were: Control siRNA (5′-UUCUCCGAACGUGUCACGU-3′), Drp1 siRNA (sense 5’-UUCAAUCCGUGAUGAGUAUGCUUUUCUUC-3’).

**Establishment of cell lines with stable expression of mitoDsRed or mitoGFP**

Stable cell lines were generated using a lentiviral system (Biosettia, cDNA-pLV01). The pLV-mitoDsRed or pLV-mitoGFP plasmid was co-transfected into HEK 293T cells together with packaging plasmids according to the manufacturer’s protocol. Three days after transfection, the cell culture was harvested and filtered. The cell-free supernatant containing the virus was then added to the target cells for transduction, followed by puromycine (1 ug/uL) selection after two days. Stable cell lines expressing either mitoDsRed or mitoGFP were confirmed under a fluorescence microscope.

**Establishment of cell lines with stable expression of shRNA**

The construction of KD cell lines followed the same procedure as that of the mitoDsRed stable line. A lentiviral-based RNAi system (Biosettia, SORT-B19) was used according to the manufacturer’s protocol. The DNA oligonucleotide sequences encoding shRNA are provided below: control LacZ (5’-GCAGTTATCTGGAAGATCAGGTTGGATCCAACCTGATCTTCCAGATAACTGC-3’), Miro1 (5’-AAAACGGGCAGAAGAAATCACCATTGGATC

CAATGGTGATTTCTTCTGCCCG-3’), MCU (5’-AAAACAATCAACTCA

AGGATGCAATTTGGATCCAAATTGCATCCTTGAGTTGATTG-3’)

**Figure S1.**

(A) HeLa cells were transfected with vector-flag/GFP (control) and DAP3-GFP/DAP3-flag for 24 h, followed by western blot analysis of mitochondria-translated proteins. (B) ARPE-19, U2OS, and HEK 293 cells were transfected with DAP3-GFP for 24 h, followed by MitoTracker staining to visualize mitochondria. Representative images of live cells are shown. (C) Quantification of mitochondria size (mean area, perimeter) and shape (circularity) per mitochondrion in (B) using ImageJ software (Particle analysis). Data are mean ± SEM. The total numbers of analyzed mitochondria are indicated below the bars. Scale bar, 10 µm. *p<0.05, **p<0.01, ***p<0.001, ****p < 0.0001.

**Figure S2.**

(A) The protein levels of Bax and Bak in the MEF cells were verified by western blot. (B,C) DAP3 does not interact with Bax and Bak. HEK293 cells co-transfected with DAP3-flag and Myc-Bax/HA-Bak were harvested after 24 h cultivation for co-immunoprecipitation (IP) using anti-Flag beads, followed by western blot analysis. (D) Fluorescent images of mitochondrial morphology in WT and Bak KO MEF cells after transfection with vector-GFP and DAP3-GFP. Mitochondria were visualized by stably expressed mito-DsRed. (E, F) Quantitative analyses of redox state in WT and Bak KO MEF cells using mitochondrial matrix-localized roGFP. Scale bar, 10 µm. *p<0.05, **p<0.01, ***p<0.001, ****p < 0.0001.

**Figure S3.**

(A) Cells in Figure 4 C, E were harvested and lysed after imaging. The protein levels of Fis1 and Drp1 were verified by western blot. (B) HEK293 cells were co-transfected with the Mfn1-flag and Mfn2-myc for 24 h. Cell lysates (containing 400 µg protein) were collected and added with 200 µg purified DAP3-His protein. After 2 h incubation, the mixtures were used for co-IP with anti-flag beads, followed by western blot analysis. (C) Purification of DAP3-His protein. DAP3 was sub-cloned into a pET42b (+) vector for protein expression. After overnight induction with 0.4 mM isopropyl β-D-1-thiogalactopyranoside (IPTG) at 20 °C, the BL21 *E. coli* was harvested and lysed for purification with nickel, cation exchange, and gel filtration column gradually. DAP3-His protein in fraction 4 and 5 after gel filtration was used in (B). BSA was used as a reference for the proteins with size of ~60 kDa. (D) HeLa cells stably expressing mito-DsRed were transfected with GFP-vector or GFP-Mfn1 for 6 h, then, a second round of transfection was performed using the DAP3-BFP plasmid. Fluorescent images were captured at 24h (upper panel). Or reversely (lower panel). Aggregated mitochondria were seen if the cell expressed GFP-Mfn1. (E) HeLa cells were transfected with DAP3-GFP or GFP-DAP3 for 24 h, followed by staining with MitoTracker to visualize mitochondria. Representative images of live cells are shown.

**Figure S4.**

(A) Cells in Figure 5A were harvested and lysed after imaging. The protein levels of Miro1 in were verified by western blot. (B) The protein levels of Mfn1 and Mfn2 in the Mfn1/2 KO MEF cells (Figure 6C) were verified by western blot. (C) The protein levels of Miro1 in the shMiro1 HeLa cells (Figure 7) were verified by western blot.

**Figure S5.**

(A) Time-lapse images in Figure 8D. Mitochondrial morphology was visualized with the mitochondria-localized GEM-GECO1 probe. (B) The protein levels of MCU in the MCU KD cells used in Figure 8G-I were verified by western blot.

**Figure S6.**

DAP3 levels in resting and inflammatory macrophages were verified by western blot.


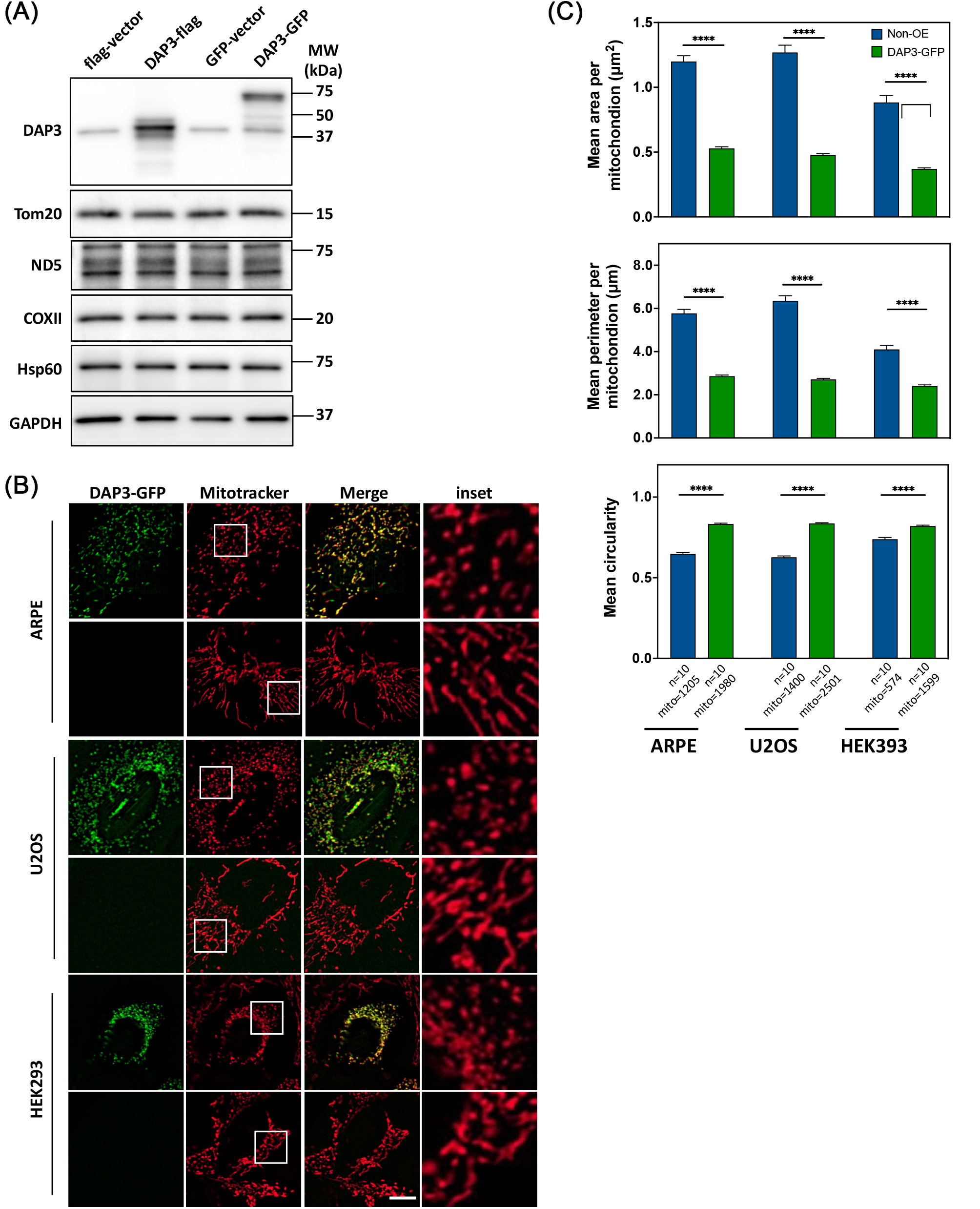


**Figure S1**


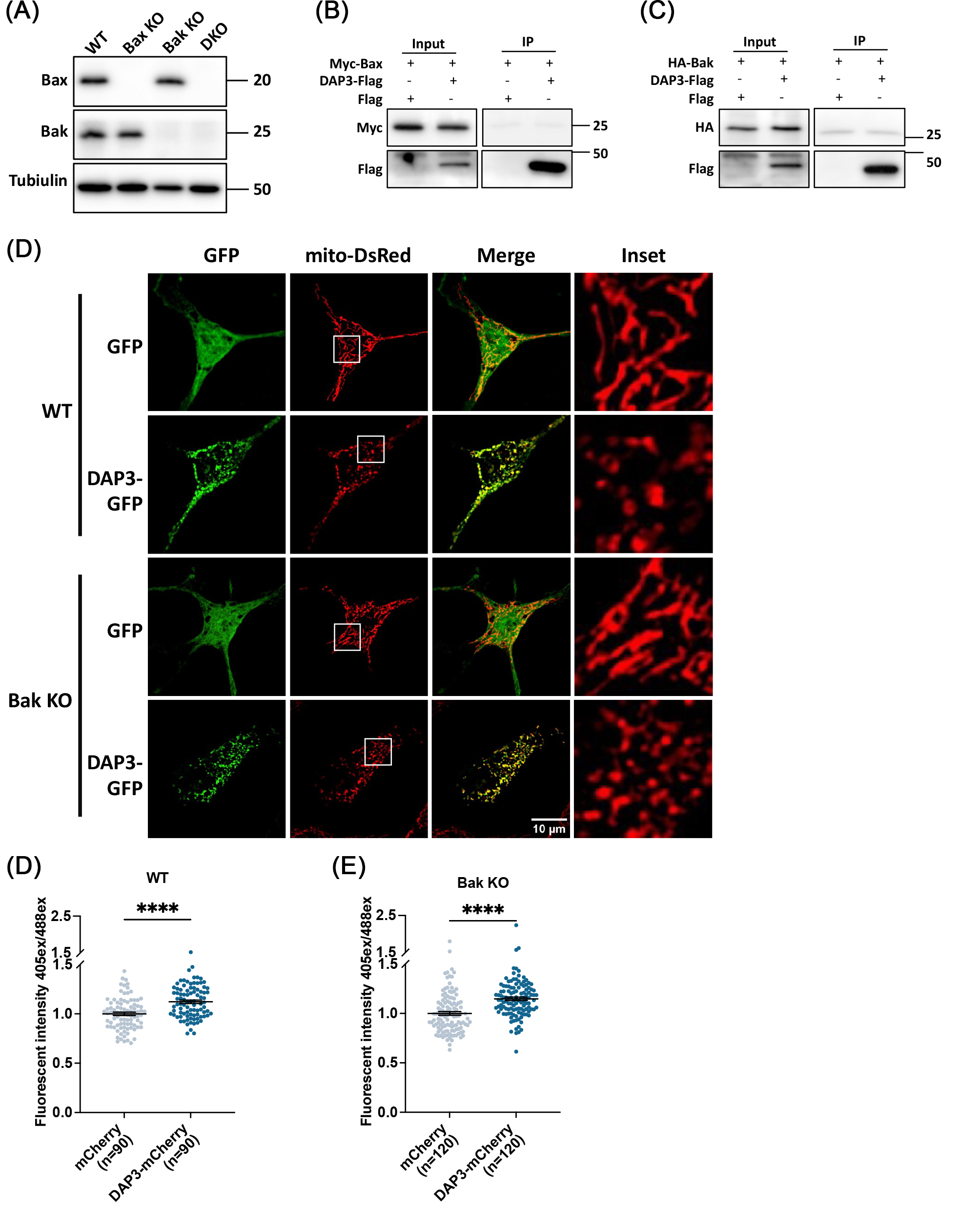


**Figure S2**


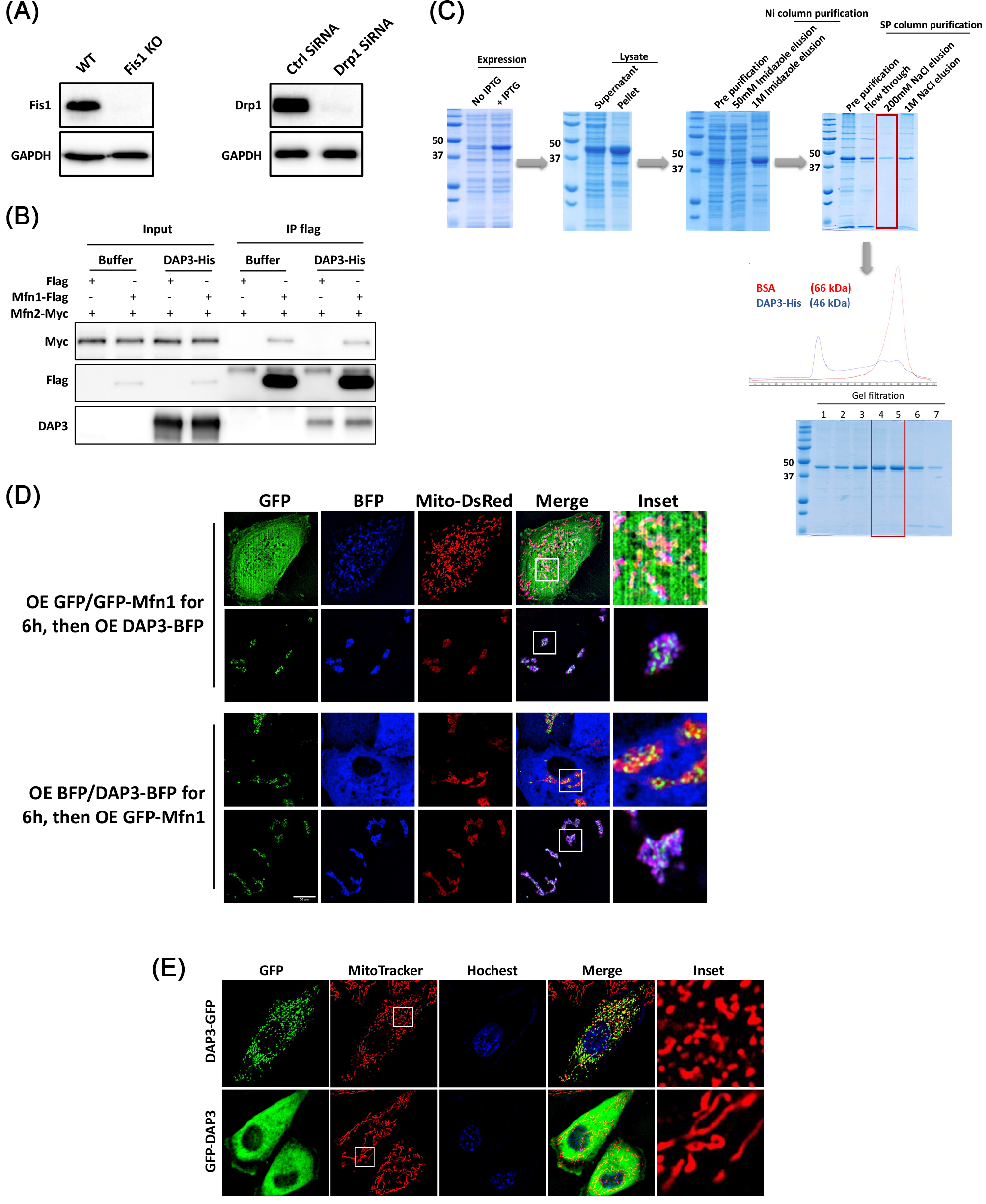


**Figure S3**


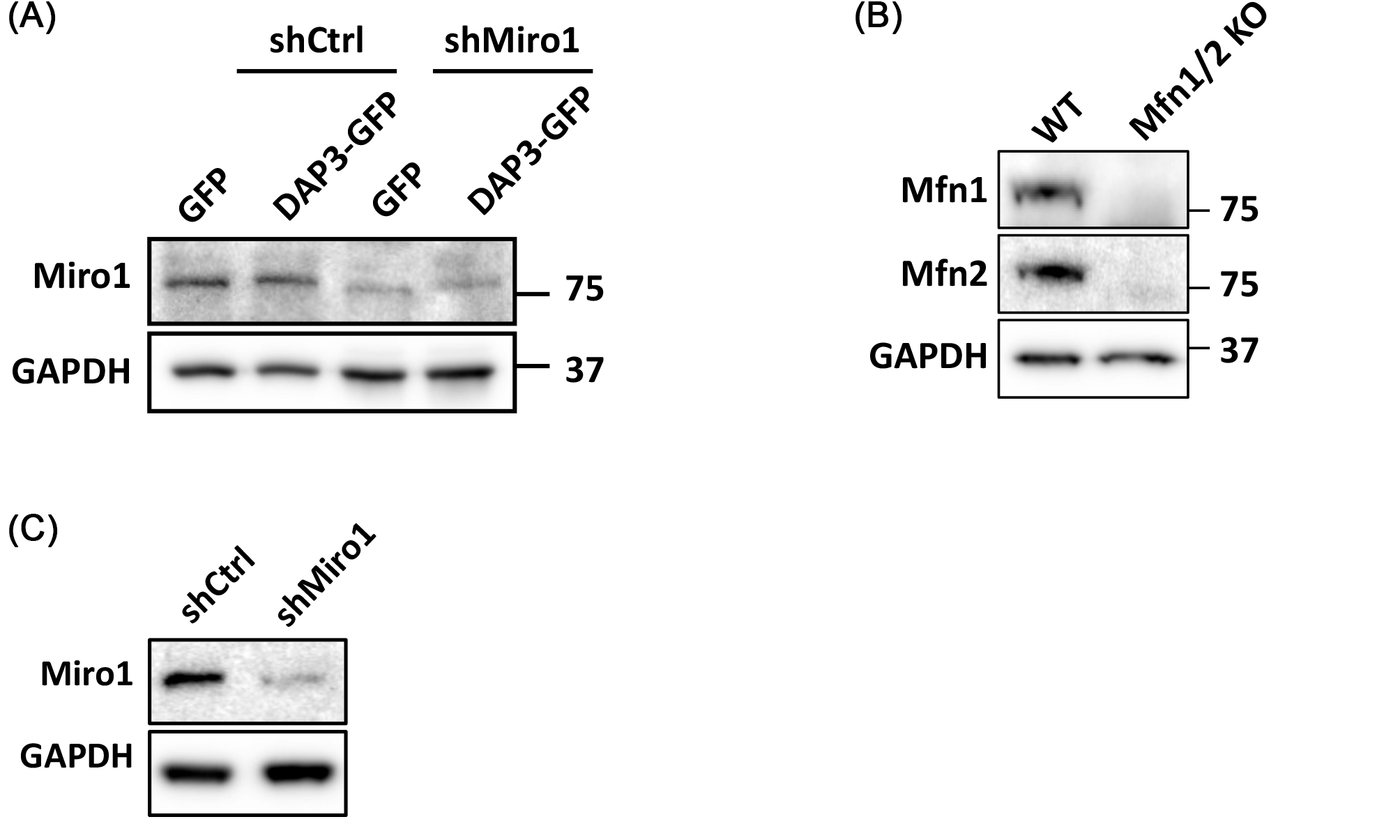


**Figure S4**


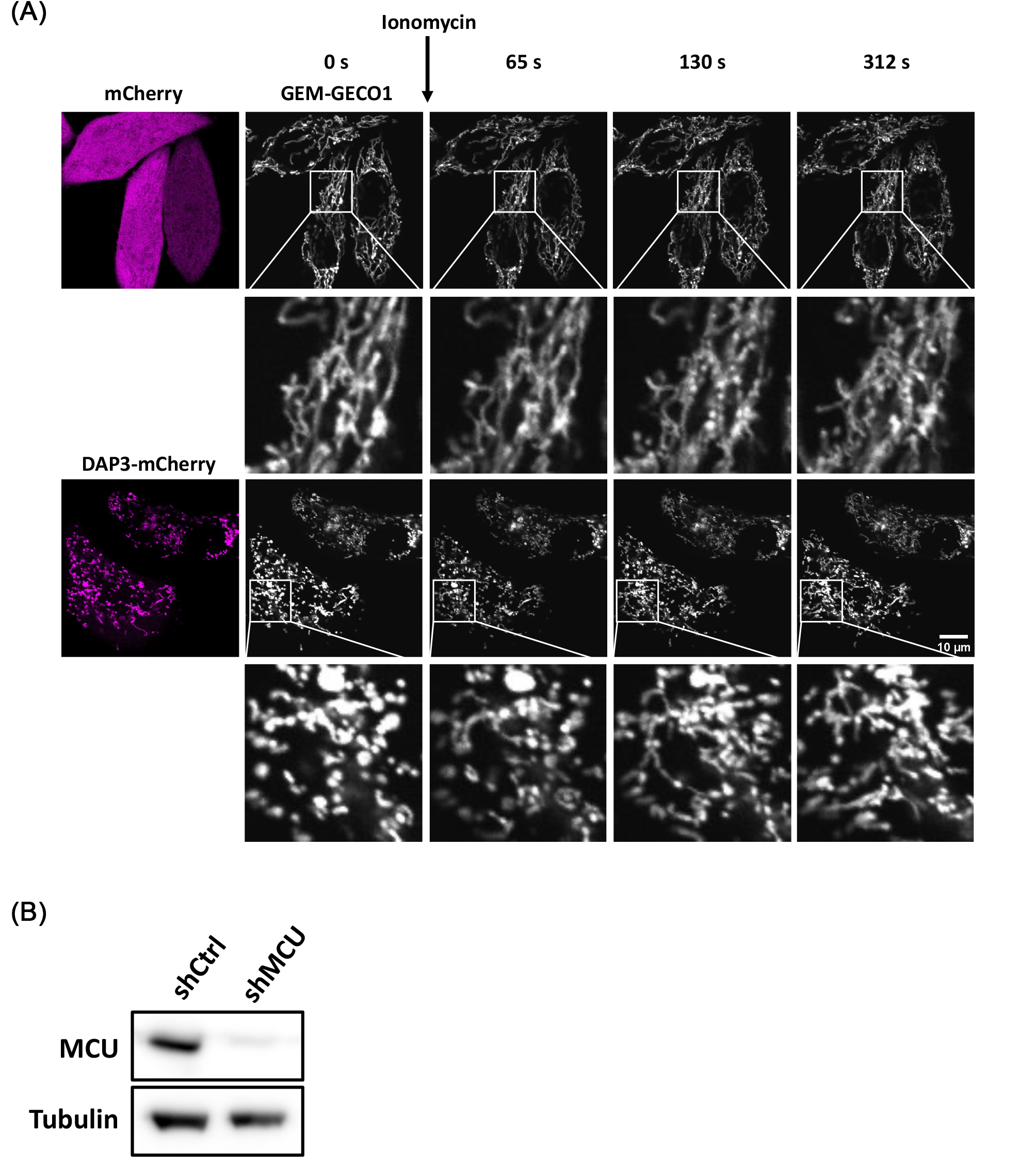


**Figure S5**

**
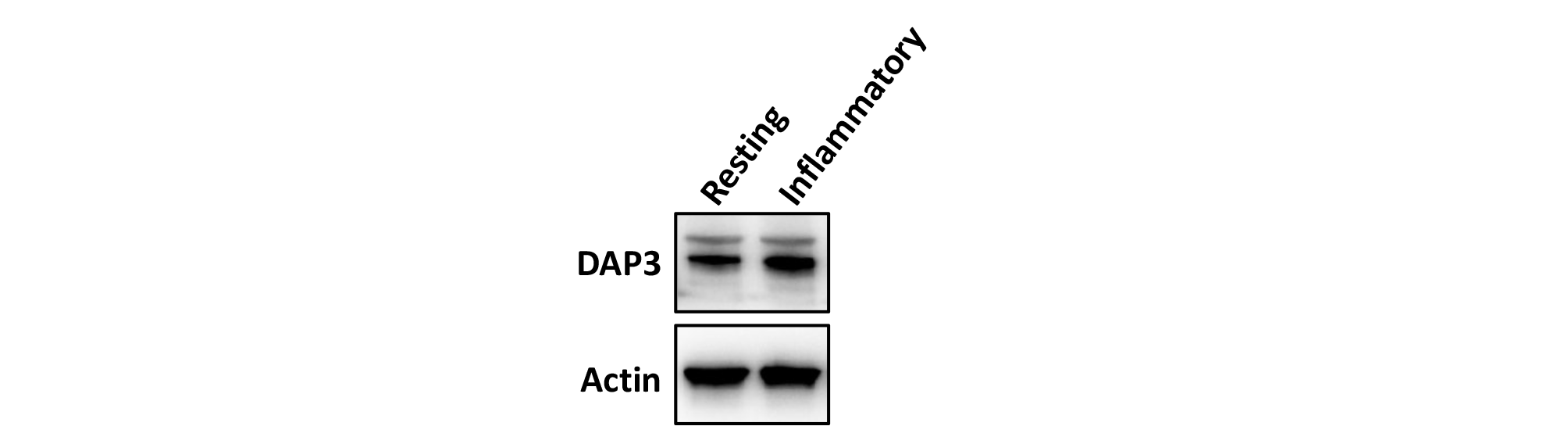
**

**Figure S6**
